# Supplementary material for: Modelling impacts of paediatric amoxicillin shortage management on pneumococcal resistance and invasive disease in Europe
Source: Nat Commun. 2026 May 19;17:6592. doi: 10.1038/s41467-026-72777-y (PMC13381528; doi:10.1038/s41467-026-72777-y)
Supplement: Supplementary file 1 — Supplementary Information [file 41467_2026_72777_MOESM1_ESM.pdf]

SUPPLEMENTARY

Figures

Figure 1. Impact of different shortage management strategies on the evolution of pneumococcal resistance, assuming a 1-year of a 50% beta-lactam shortage, depending on the value of Rcr^{init}.

Figure 2. 1-year impact of a 50% beta-lactam shortage on antibiotic resistance and the incidence of invasive pneumococcal disease (IPD), depending on the antibiotic shortage management strategy.

Figure 3. Multivariate sensitivity analysis of four main outcomes : partial rank correlation coefficient (PRCC) for the main model parameters under the four scenarios.

Figure 4. Best antibiotic allocation strategy depending on resistance level initial condition and parameters values.

Figure 5. Best antibiotic allocation strategy depending on resistance level initial condition and MIC distribution.

Figure 6. Sensitivity analysis of θ competition parameter.

Figure 7. Variation in S. pneumoniae resistance proportion and invasive disease incidence among children under five years of age, across different shortage management strategies following a 1-year period of 50% shortage compared to no-shortage for different relative fitness values.

Figure 8. Correlation matrix between initial conditions on country-specific pharmaco-epidemiological context and outcome variation after 1-year.

Figure 9. Absolute variation in *S. pneumoniae* invasive disease incidence among children under five years of age for each resistance level to both antibiotics.

Figure 10. Results of calibration of the model to data on the impact of amoxicillin-clavulanate.

Figure 11. Results of calibration of the model to data on the impact erythromycin.

Figure 12. Amoxicillin treatment induced decolonization rate for different shortage levels.

Figure 13. Association between antibiotic consumption frequency and associated resistance across 20 European countries.

Tables

Table 1. Summary of the model parameters for the estimation of antibiotic-induced decolonization parameters.

Table 2. Parameters of the model in a French and European context.

Table 3. Country-specific pharmaco-epidemiological parameters.

Descriptions

Description 1. Absolute variation in *S. pneumoniae* invasive disease incidence, considering a 1-year 50% beta-lactam shortage in the French context

Description 2. Calculation explanation of the Number of DDD.

Equations

Equation 1. Transmission model equations of pneumococcus colonisation.

I. Figures

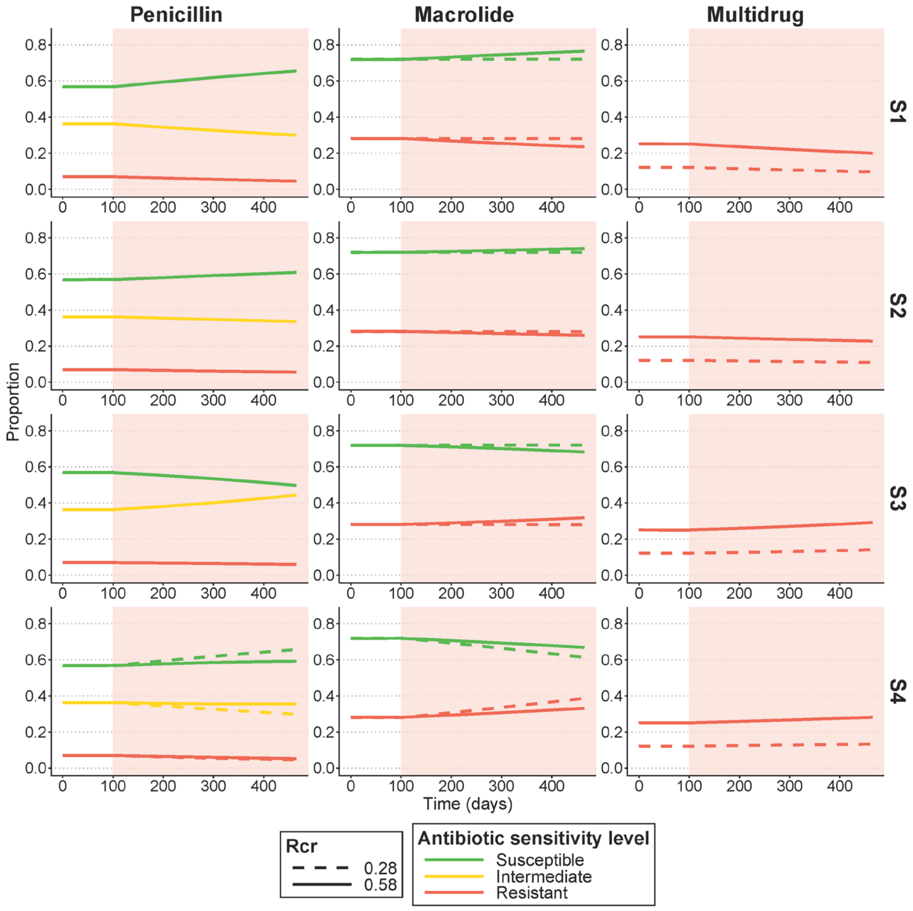

Supplementary Figure 1. Impact of different shortage management strategies on the evolution of pneumococcal resistance, assuming a 1-year of a 50% beta-lactam shortage, depending on the value of Rcr^{init}.

Rcr^{init} corresponds to the initial proportion of macrolide-resistant strains among penicillin non-susceptible strains (i.e., multi-resistance). If $Rcr^{init} = MRSP^{init} = 0.28$, both resistance are independent and multi-resistance is not higher than expected by chance; if $Rcr^{init} > MRSP^{init}$ $(eg. Rcr^{init} = 0.58)$, penicillin-resistance and macrolide-resistance are not independent.

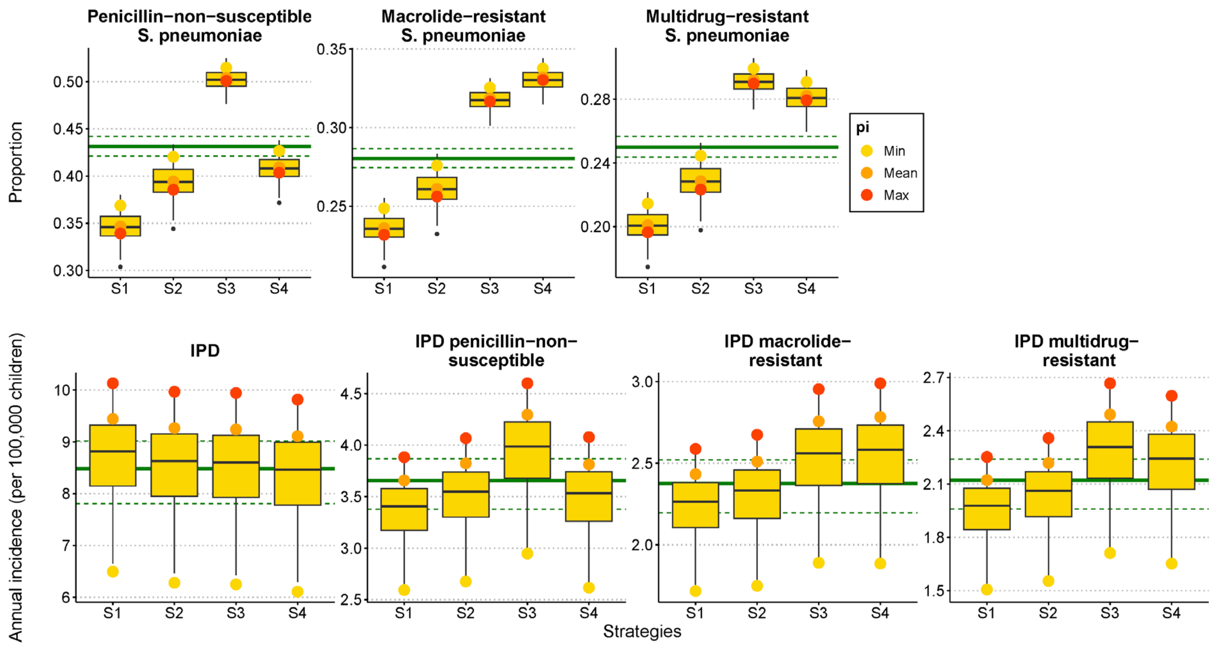

Supplementary Figure 2. 1-year impact of a 50% beta-lactam shortage on antibiotic resistance and the incidence of invasive pneumococcal disease (IPD), depending on antibiotic shortage management strategy in the French context.

Assuming a 50% shortage, 4 strategies are explored: reducing beta-lactam consumption frequency (S1), reducing beta-lactam treatment duration (S2), reducing beta-lactam daily dose (S3), switching from beta-lactam to macrolide prescription (S4). Seven outcomes are provided. The top row shows the relative variation in the proportion of antibiotic-resistant strains compared with no shortage : penicillin-non-susceptible *S. pneumoniae* (PNSP) proportion, macrolide-resistant *S. pneumoniae* (MRSP) proportion, multidrug-resistant *S. pneumoniae* (MDRSP) proportion. The bottom row shows the relative variation of invasive pneumococcal disease (IPD) incidence per 100,000 children per year compared with no shortage, overall incidence (IPD), penicillin-non-susceptible IPD incidence (IPD_{PNSP}), macrolide-resistant IPD incidence (IPD_{MRSP}), and multidrug-resistant IPD incidence (IPD_{MDRSP}). The results are shown accounting for an uncertainty in the duration of carriage and the initial carriage prevalence. For each outcome and strategy, boxplots provide the median (black horizontal line), the interquartile range (upper and lower bounds of the box), and minimum and maximum values (whiskers) across n = 200 simulations obtained by Latin Hypercube Sampling of two input parameters: duration of carriage (triangular distribution: min = 32, central value = 43, max = 51 days) and initial carriage prevalence (triangular distribution: min = 0.35, central value = 0.52, max = 0.56). For each outcome, the bold green line depicts the median value obtained in the baseline scenario without beta-lactam shortage, and the dotted lines the associated interquartile range across the same n = 200 simulations. Additionally, the yellow, orange and red dots correspond to the outcome values for the minimum (p=0.35), average (p=0.52) and maximum (p=0.56) carriage prevalence values, with the carriage duration fixed at 43 days.

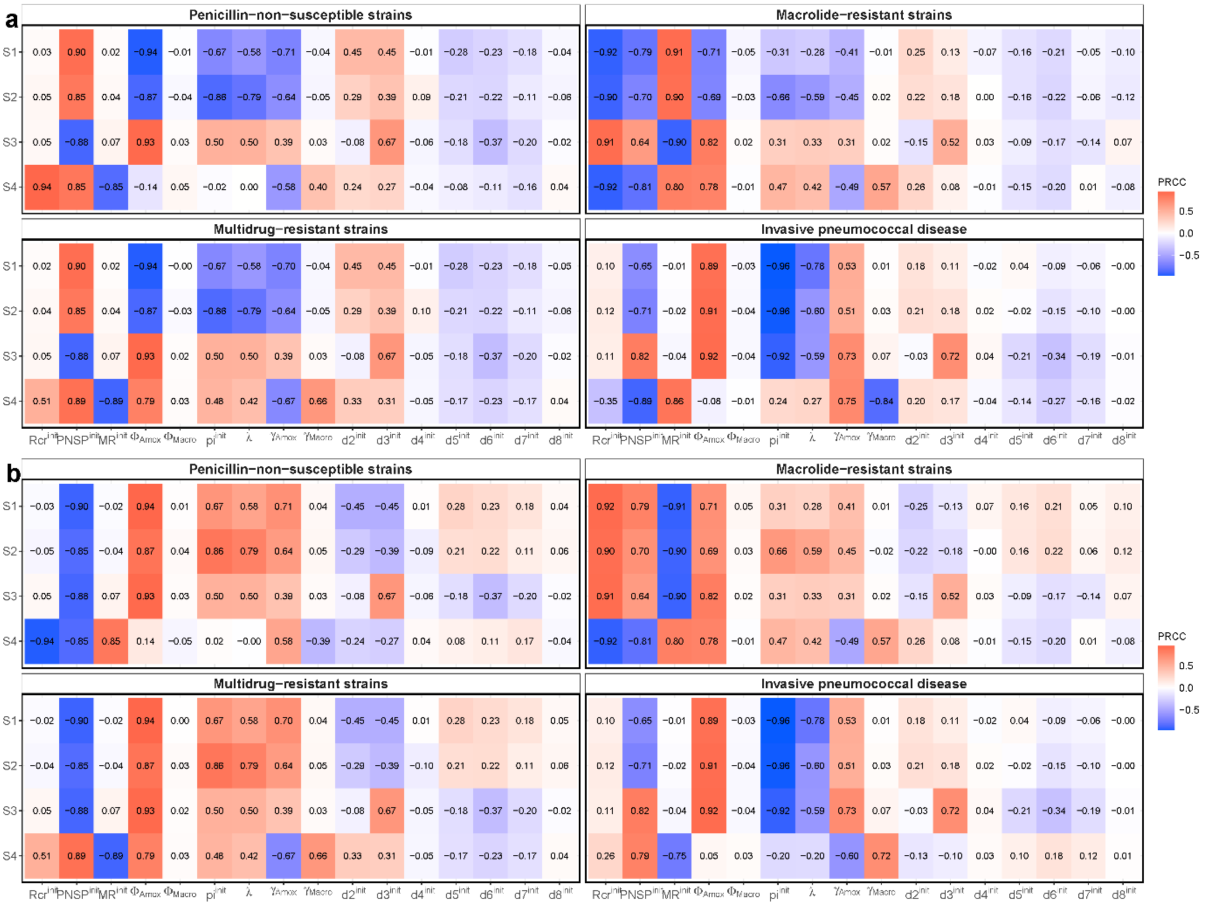

Supplementary Figure 3. Multivariate sensitivity analysis of four main outcomes : partial rank correlation coefficient (PRCC) for the main model parameters under the four scenarios.

a) PRCC of the variation of the four outcomes of interest after a 1-year period of 50% shortage, under the different proposed shortage management strategies. **b)** PRCC of the absolute value of the variation of the four outcomes of interest after a 1-year period of 50% shortage, under the different proposed shortage management strategies. A red cell indicates parameters for which a parameter increase is associated with an increase in the variation (or absolute value of the variation) of the outcome. Blue cells indicate parameters for which a parameter increase is associated to a decrease in the variation (or absolute value of the variation). Using the absolute value allowed us to focus on the amplitude in these variations, irrespective of their sign.

In a context of shortage, allocation strategies led to a decrease or an increase of the outcome. Then, red box indicates increases in the outcome value for positive initial values or decreases in the outcome for negative initial values. We chose to perform multivariate sensitivity analysis using the absolute value of the variations to focus on the amplitude in these variations, irrespective of their sign.

Multivariate sensitivity analysis on the absolute value of the variations show that the parameters that most impact our predictions (parameters with PRCC>0.6 or PRCC<-0.6) are Rcr^{init} $PNSP^{init}$, MR^{init}, Φ^{Amox} , p^{init}, λ, γ^{Amox} γ^{Macro} and $d3^{init}$. Three parameter influence patterns across explored strategies can be highlighted: (1) parameters with strong correlations across all strategies with a given outcome, such as Φ^{Amox} and λ which consistently correlate positively with IPD variation, whereas pi^{init} consistently correlates negatively; (2) parameters with strong correlations but varying effects across strategies for a same outcome. An example is $PNSP^{init}$

which is inversely correlated with IPD variation under S1 and S2, while it is positively correlated under S3 and S4; and finally (3) parameters for which the impact on a given outcome depends on the strategy. Examples are MR^{init} and γ^{Macro} which show respectively a negative and a positive correlation with IPD when applying S4 whereas no impact in the IPD evolution could be observed with S1, S2 and S3.

This analysis highlights overall the parameters that should be considered when making the choice of optimal strategy in a beta-lactam shortage context, which are those meeting criteria (2) or (3). Specifically, key parameters are Rcr^{init}, MR^{init}, p^{init} and λ for penicillin-non-susceptible strains; Rcr^{init}, $PNSP^{init}$ and MR^{init} for macrolide-resistant strains; and $PNSP^{init}$, MR^{init}, γ^{Amox}, γ^{Macro} for multidrug-resistant strains and severe infection incidence.

a)

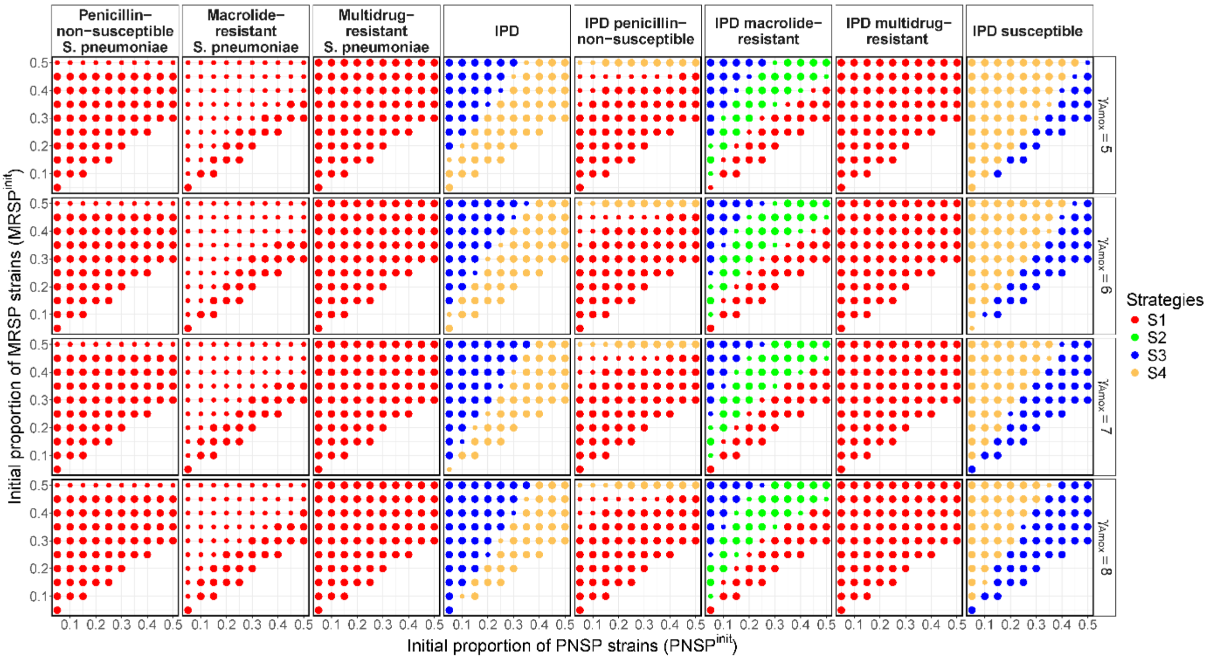

Penicillin–non–susceptible S. pneumoniae
Macrolide–resistant S. pneumoniae
Multidrug–resistant S. pneumoniae
IPD
IPD penicillin–non–susceptible
IPD macrolide–resistant
IPD multidrug–resistant
IPD susceptible
$\gamma_{Amox} = 5$
$\gamma_{Amox} = 6$
$\gamma_{Amox} = 7$
$\gamma_{Amox} = 8$
Initial proportion of MRSP strains ($MRSP^{init}$)
Initial proportion of PNSP strains ($PNSP^{init}$)
0.1 0.2 0.3 0.4 0.5
Strategies
S1
S2
S3
S4

b)

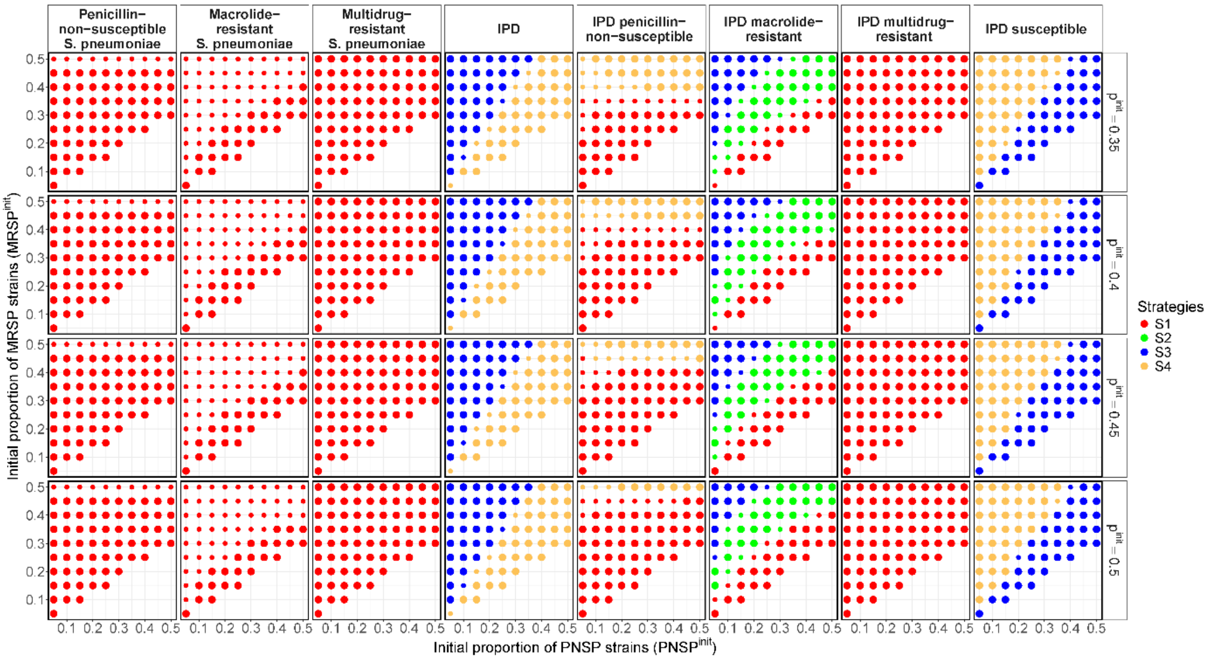

Penicillin–non–susceptible S. pneumoniae
Macrolide–resistant S. pneumoniae
Multidrug–resistant S. pneumoniae
IPD
IPD penicillin–non–susceptible
IPD macrolide–resistant
IPD multidrug–resistant
IPD susceptible
$p^{init} = 0.35$
$p^{init} = 0.4$
$p^{init} = 0.45$
$p^{init} = 0.5$
Initial proportion of MRSP strains ($MRSP^{init}$)
Initial proportion of PNSP strains ($PNSP^{init}$)
0.1 0.2 0.3 0.4 0.5
Strategies
S1
S2
S3
S4

c)

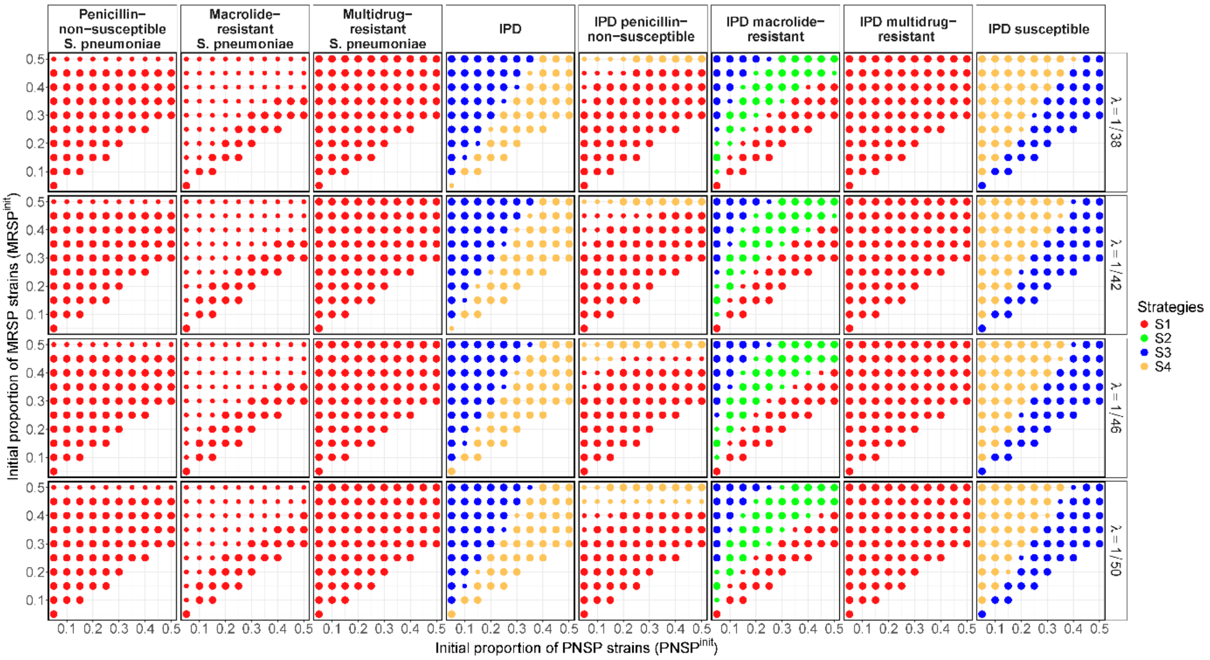

Supplementary Figure 4. Best antibiotic allocation strategy depending on resistance level initial condition and parameters values.

Recommended antibiotic shortage management strategy depending on the initial proportions of penicillin -non-susceptible strains ($PNSP^{init}$) (x-axis), macrolide-resistant strains (MR^{init}) (y-axis) and the value of **a)** duration of exposure (γ^{Amox}) **b)** carriage prevalence (p^{init}) and **c)** duration of colonization($1/\lambda$). For each initial condition combination, the strategy minimizing each outcome is depicted with a coloured dot. A strategy is considered better (indicated by a large dot) when its value differs by more than 5% from the others for proportion-based outcomes, or by at least 1 IPD/1 million children/year for incidence-based outcomes.

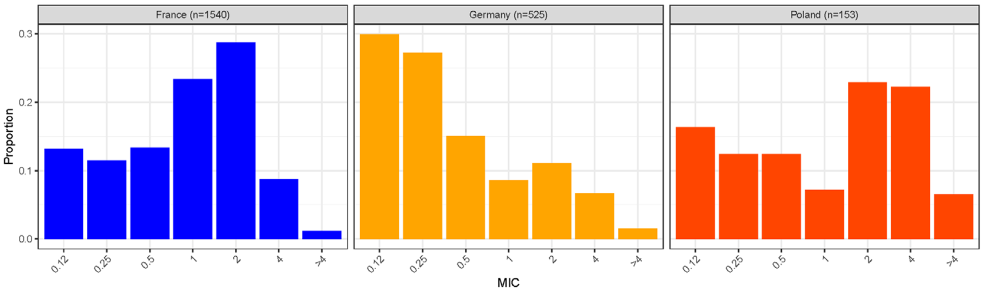
France (n=1540)
Germany (n=525)
Poland (n=153)
Proportion
0.0
0.1
0.2
0.3
0.12
0.25
0.5
1
2
4
>4
MIC

a)

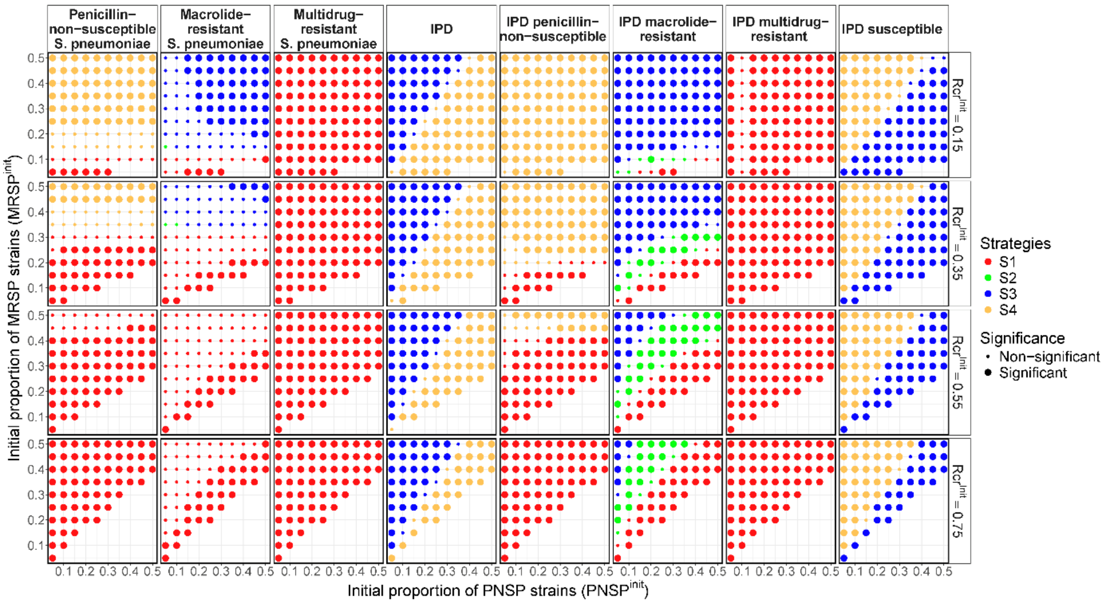
Penicillin−non−susceptible S. pneumoniae
Macrolide−resistant S. pneumoniae
Multidrug−resistant S. pneumoniae
IPD
IPD penicillin−non−susceptible
IPD macrolide−resistant
IPD multidrug−resistant
IPD susceptible
Rcr^init = 0.15
Rcr^init = 0.35
Rcr^init = 0.55
Rcr^init = 0.75
Initial proportion of MRSP strains (MRSP^init)
Initial proportion of PNSP strains (PNSP^init)
0.1 0.2 0.3 0.4 0.5
Strategies
S1
S2
S3
S4
Significance
Non−significant
Significant

b)

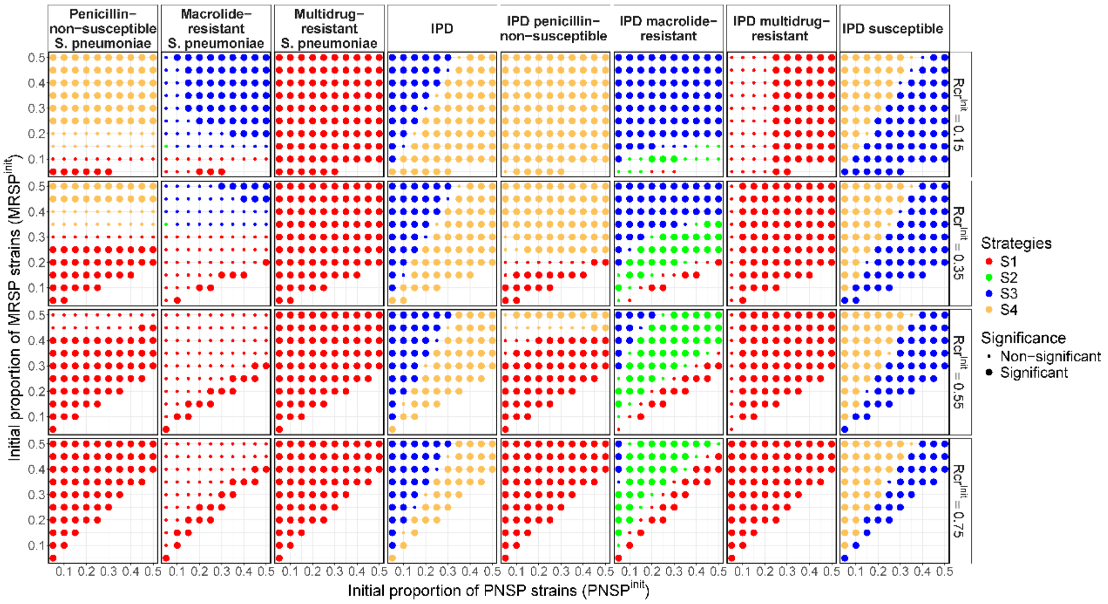
Penicillin−non−susceptible S. pneumoniae
Macrolide−resistant S. pneumoniae
Multidrug−resistant S. pneumoniae
IPD
IPD penicillin−non−susceptible
IPD macrolide−resistant
IPD multidrug−resistant
IPD susceptible
Rcr^init = 0.15
Rcr^init = 0.35
Rcr^init = 0.55
Rcr^init = 0.75
Initial proportion of MRSP strains (MRSP^init)
Initial proportion of PNSP strains (PNSP^init)
0.1 0.2 0.3 0.4 0.5
Strategies
S1
S2
S3
S4
Significance
Non−significant
Significant

c)

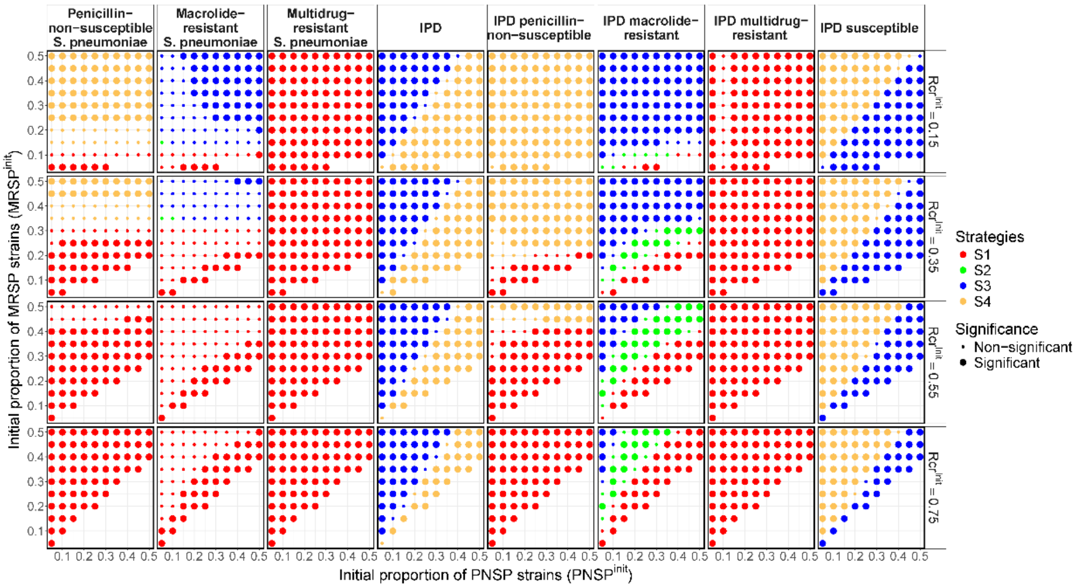

Supplementary Figure 5. Best antibiotic allocation strategy depending on resistance level initial condition and MIC distribution.

In all figures, the selected strategy is shown for different assumptions on initial proportion of penicillin-non-susceptible Streptococcus pneumoniae ($PNSP^{init}$) (x-axis), macrolide-resistant Streptococcus pneumoniae ($MRSP^{init}$) (y-axis), the proportion of macrolide-resistant strains among penicillin-non-susceptible strains (Rcr^{init}) and depending on the initial MIC distribution: **a)** French MIC distribution, **b)** German MIC distribution, and **c)** Polish MIC distribution.

Penicillin MIC values for *S. pneumoniae* by country were sourced from the ATLAS surveillance database for the period 2004-2023.

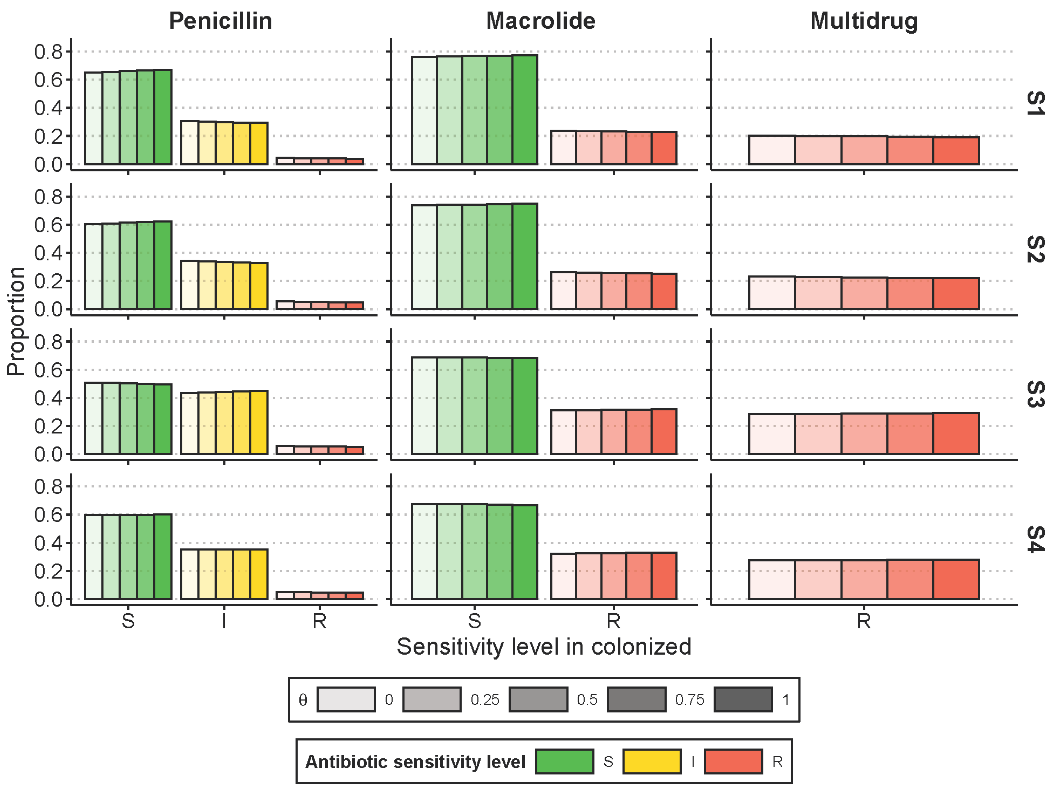

Supplementary Figure 6. Sensitivity analysis of θ competition parameter on *S. pneumoniae* resistance, depending on the antibiotic shortage management strategy.

We observed minimal impact from varying θ between 0 and 1 on the resistance outcome values. In fact, changes to the θ parameter primarily affected the fitted values of the fitness cost parameter. Increasing θ (which allows for more opportunities for replacement) resulted in a decrease in the fitted fitness cost, reflected by an increase in the parameter value.

Theta/Calibrated values	f_{CMI1}^{Amox}	f_{CMI2}^{Amox}	f_{CMI3}^{Amox}	f_{CMI4}^{Amox}	f_{CMI5}^{Amox}	f_{CMI6}^{Amox}	f_{CMI7}^{Amox}	f_{CMI8}^{Macro}	β
0	0.998	0.987	0.917	0.884	0.884	0.884	0.884	0.991	0.055
0.25	0.998	0.988	0.934	0.905	0.905	0.905	0.905	0.993	0.055
0.50	0.999	0.990	0.941	0.918	0.918	0.918	0.918	0.994	0.055
0.75	0.999	0.992	0.947	0.927	0.927	0.927	0.927	0.995	0.055
1	0.999	0.993	0.952	0.934	0.934	0.934	0.934	0.995	0.055

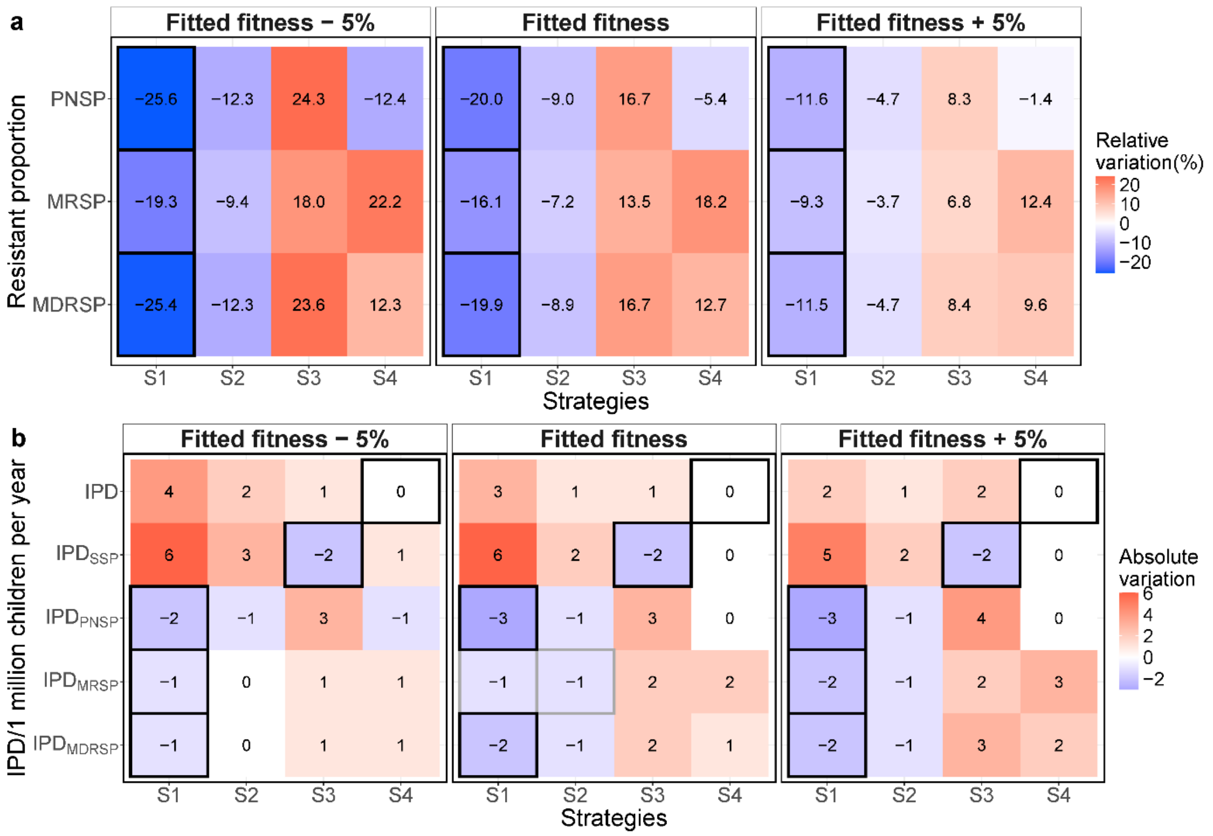

Supplementary Figure 7. Variation in S. pneumoniae resistance proportion and invasive disease incidence among children under five years of age, across different shortage management strategies following a 1-year period of 50% shortage compared to no-shortage for different relative fitness values.

4 strategies are explored: reducing beta-lactam consumption frequency (S1), reducing beta-lactam treatment duration (S2), reducing beta-lactam daily dose (S3), switching from beta-lactam to macrolide prescription (S4). The optimal strategy is framed. a) Three resistance proportion outcomes predicted: penicillin-non-susceptible S. pneumoniae (PNSP) proportion; macrolide-resistant S. pneumoniae (MRSP) proportion; and multidrug-resistant S. pneumoniae (MDRSP) proportion. For each outcome, black squares indicate the predicted optimal strategy for which the predicted proportion differs by more than 5% from all the other strategies, while grey lines indicate the best strategy, when the difference is lower. b) Five IPD outcomes are predicted: overall incidence (IPD);susceptible IPD incidence (IPD_{SSP}); penicillin-non-susceptible IPD incidence (IPD_{PNSP}) incidence; macrolide-resistant IPD incidence (IPD_{MRSP}); and multidrug-resistant IPD incidence (IPD_{MDRSP}). Black lines indicate the optimal strategy for which the predicted number of IPD cases differs from at least 1 case per million children per year from all the other strategies, while grey lines indicate the best strategy, but with a lower difference. The baseline tested relative fitness values are [1, 0.999, 0.992, 0.942, 0.919, 0.919, 0.919, 0.919], varied by ±5%.

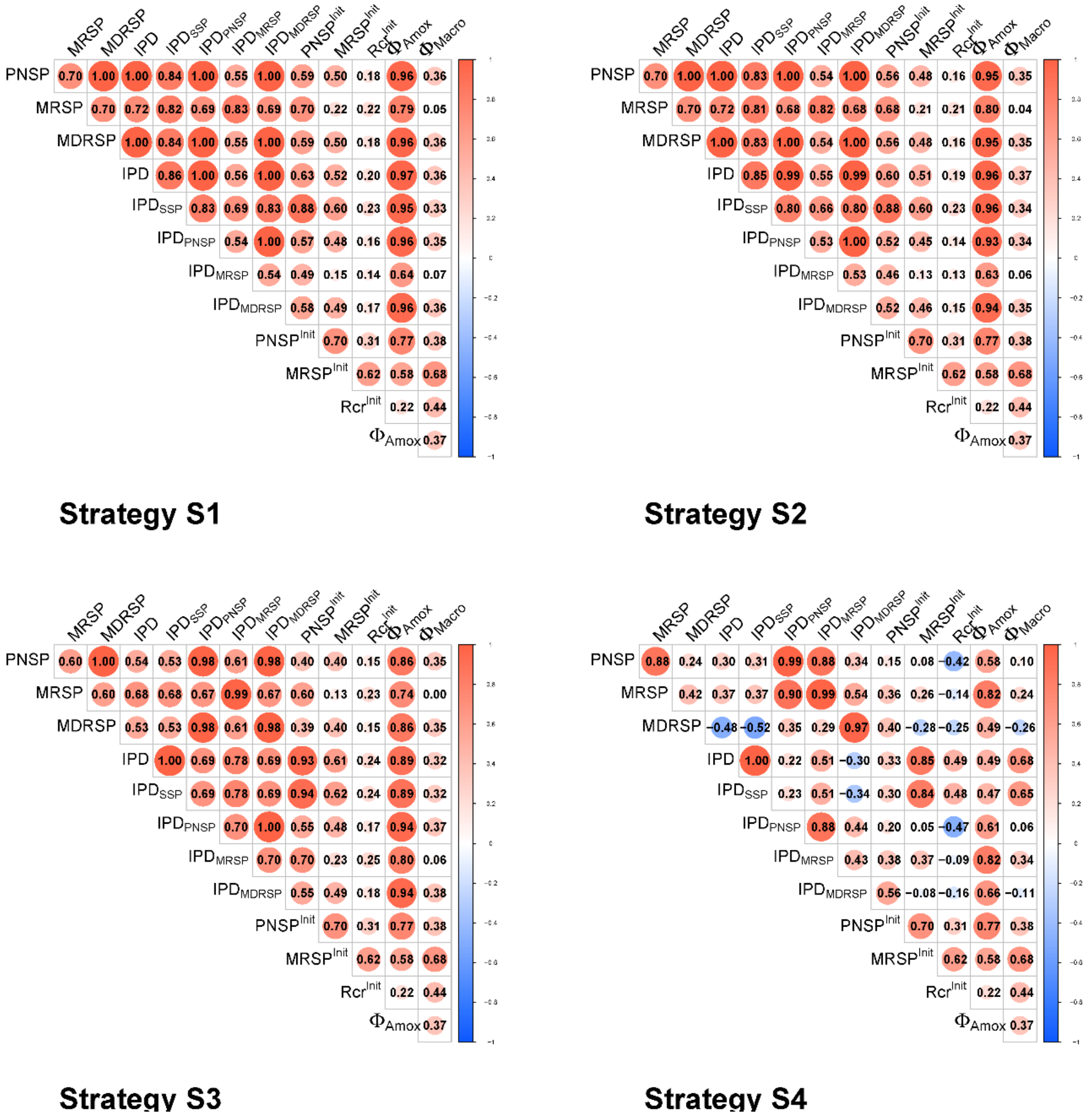

Supplementary Figure 8. Correlation matrix between initial conditions on country-specific pharmaco-epidemiological context and outcome variation after 1-year.

The outcome measured after 1-year of shortage are $PNSP$, $MRSP$, $MDRSP$, IPD, IPD_{PNSP}, IPD_{MRSP} and IPD_{MDRSP} absolute value of the relative variation between shortage and no-shortage. Using the absolute value allowed us to focus on the amplitude in these variations, irrespective of their sign. The parameters that vary according to the different country-specific context are $PNSP^{init}$, $MRSP^{init}$, Rcr^{init}, Φ^{Amox} and Φ^{Macro}.

This correlation matrix is a univariate analysis that measures the strength and direction of linear relationships between two variables at a time. This analysis does not account for the influence of other variables, which can lead to misleading interpretations if confounding variables are present. This explains why the correlations found in the PRCC analysis, which is multivariate, may differ. Indeed, the PRCC determines the correlation between two variables while controlling for the influence of other variables.

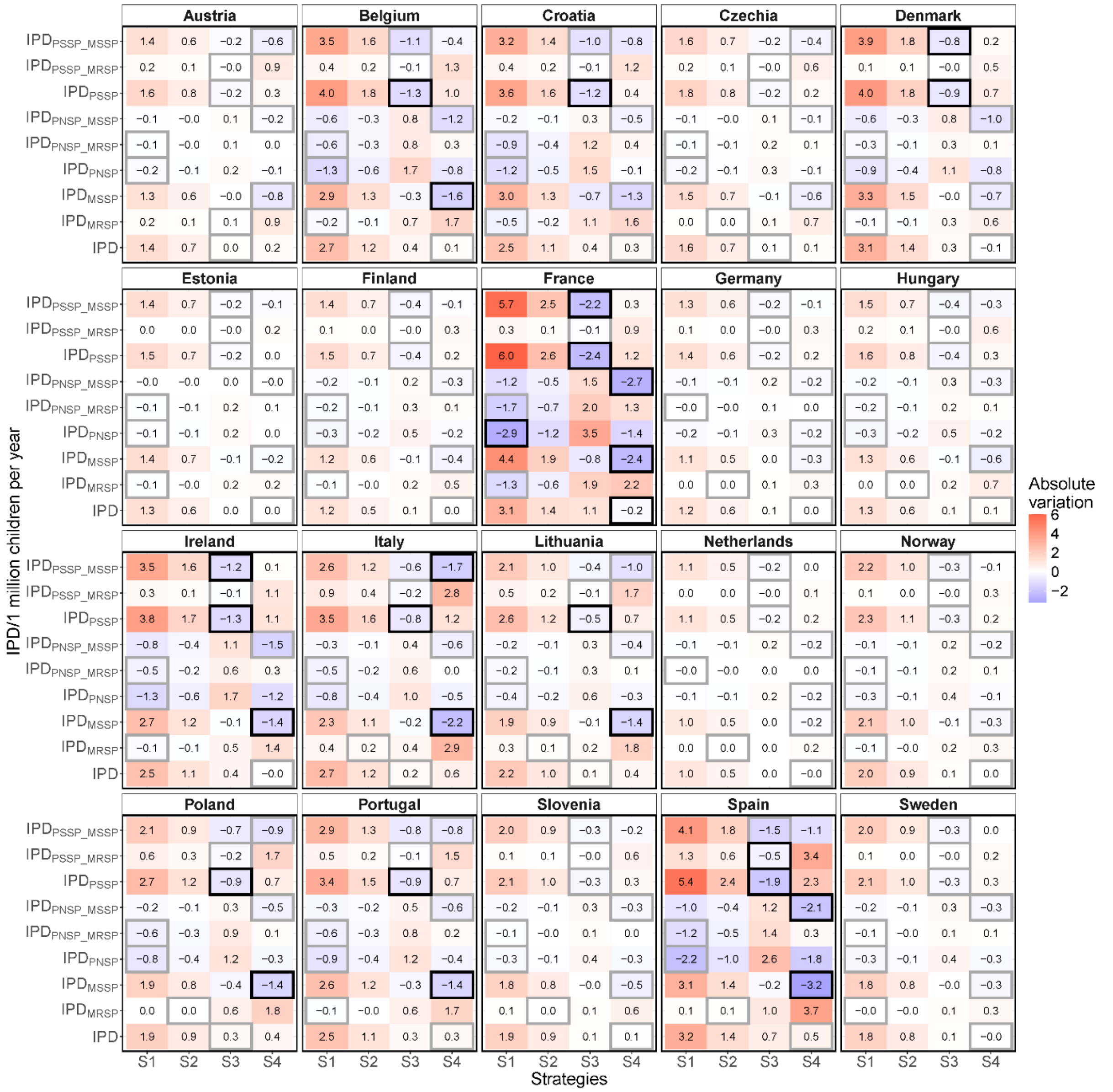

Supplementary Figure 9. Absolute variation in *S. pneumoniae* invasive disease incidence among children under five years of age for each resistance level to both antibiotics.

Nine outcomes on the absolute variation of invasive pneumococcal disease incidence compared with no shortage are provided for each resistance levels to the two antibiotics, from top to bottom : penicillin-susceptible and macrolide-susceptible IPD incidence (IPD_{PSSP_MS}), penicillin-susceptible and macrolide-resistant IPD incidence (IPD_{PSSP_MR}), penicillin-susceptible IPD incidence (IPD_{PSSP}) incidence, penicillin-non-susceptible and macrolide-susceptible IPD incidence (IPD_{PNSP_MS}), penicillin-non-susceptible and macrolide-resistant IPD incidence (IPD_{PNSP_MR}), penicillin-non-susceptible IPD incidence (IPD_{PNSP}) incidence, macrolide-susceptible IPD incidence (IPD_{MS}), macrolide-resistant IPD incidence (IPD_{MR}), and overall IPD incidence (IPD_{MDR}).

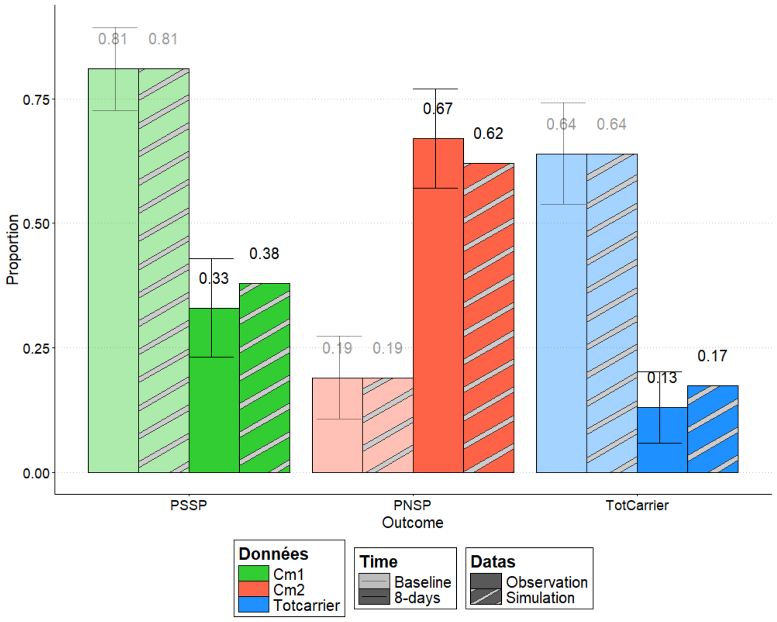

Supplementary Figure 10. Results of calibration of the model to data on the impact of amoxicillin-clavulanate (5.7mg/kg, in three divided doses, for seven days) against *S. pneumoniae* carriage prevalence (TotCarrier) and penicillin-non-susceptible *S. pneumoniae* (PNSP) proportion, observed in 162 children aged 6-35 months, 1 day after the end of the treatment[1].

We performed these calibrations by minimizing the least squares error between model predictions of pneumococcal carriage 1 day after the end of the treatment running the Limited-memory BFGS (L-BFGS) optimization algorithm that approximates the Broyden-Fletcher-Golfarb-Shanno algorithm using box constraints[2], limiting at 1 the antibiotic-induced decolonization rate on susceptible strains per days.

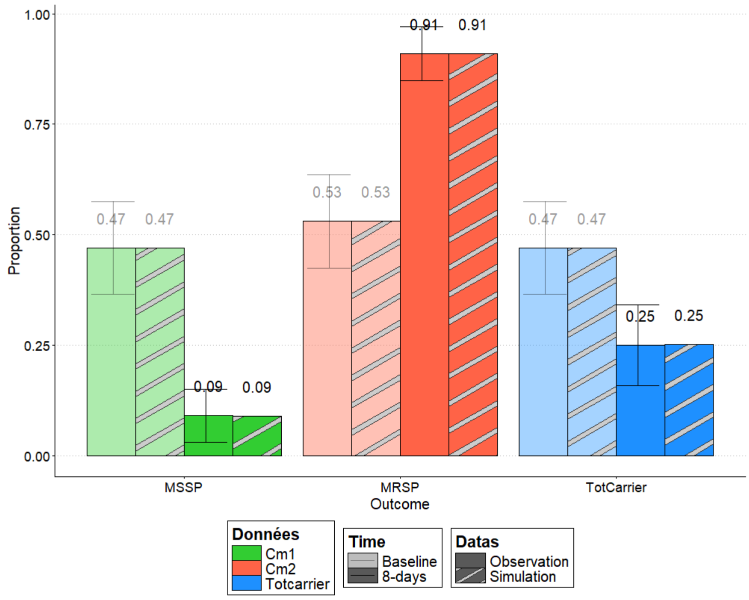

Supplementary Figure 11. Results of calibration of the model to data on the impact of erythromycin/sulfisoxazole (50mg, 150mg, in 3 divided doses, for eight days) against *S. pneumoniae* carriage prevalence (TotCarrier) and macrolide-resistant *S. pneumoniae* (MRSP) proportion, observed in 102 children aged 6-35 months, 2 days after the end of the treatment[3].

We performed these calibrations by minimizing the least squares error between model predictions of pneumococcal carriage 1 day after the end of the treatment running the Limited-memory BFGS (L-BFGS) optimization algorithm that approximates the Broyden-Fletcher-Golfarb-Shanno algorithm using box constraints[2], limiting at 1 the antibiotic-induced decolonization rate on susceptible strains per days.

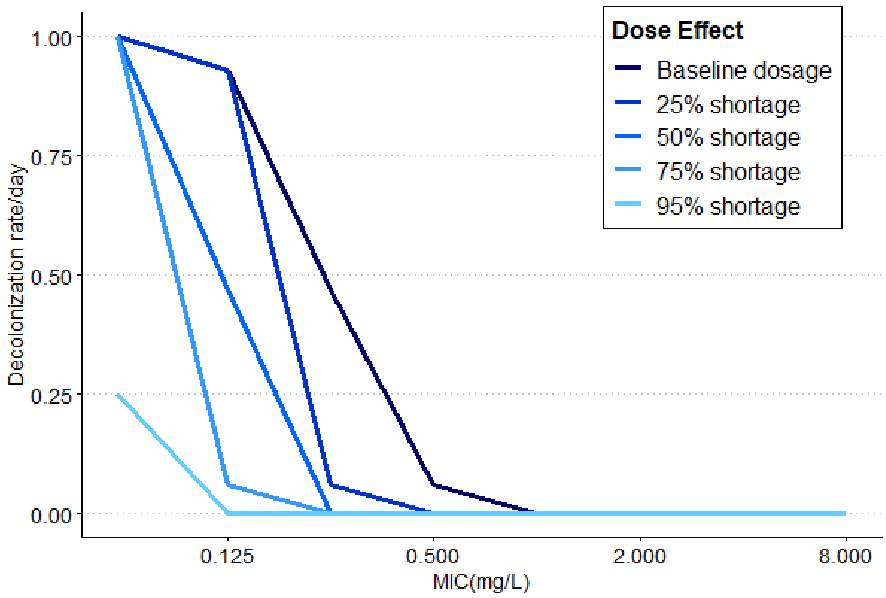

Supplementary Figure 12. Amoxicillin treatment induced decolonization rate for different shortage levels. Calibrated to follow observed change in the %ft>MIC across different MIC levels when the daily dose is reduced (de Velde, F. *et al.* Non-linear absorption pharmacokinetics of amoxicillin: consequences for dosing regimens and clinical breakpoints[4]).

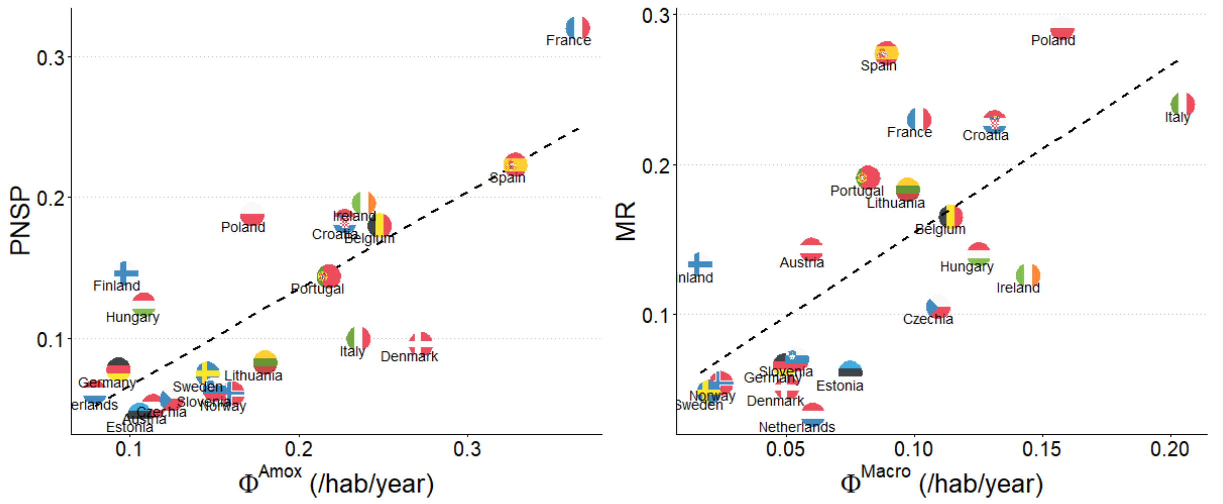

Supplementary Figure 13. Association between antibiotic consumption frequency and associated resistance across 20 European countries. The proportion of penicillin-non-susceptible *S. pneumoniae* ($PNSP$) in carriers, the proportion of macrolide-resistant *S. pneumoniae* ($MRSP$) in carriers, the frequency of consumption of both beta-lactams (Φ^{Amox}) and macrolides (Φ^{Macro}) per person per year. Specifically, data on beta-lactam and macrolide consumption were obtained through the European Surveillance of Antimicrobial Consumption Network (ESAC-Net)5, using 2021 data. Data on pneumococcal resistance were sourced from the European Antimicrobial Resistance Surveillance Network (EARS-Net)6, also using 2021data.

II. Tables

	Amoxicillin-clavulanate (40 and 5.7mg/kg/d, respectively, in two divided doses)	Erythromycin/sulfisoxazole (50 and 150mg/kg/d, respectively, in three divided doses)	Sources
**Descriptive data on the clinical studies on antibiotic exposure impact**			
Population size	162	102	Amoxicillin study[1] Erythromycin *study*[3]
Age of the population	6-35 months	3-36 months	
**Literature-derived parameters used for model calibration**			
Duration of colonization ($1/\lambda$) (days)	43	43	Högberg et al.[7] Ekdahl et al.[8]
Antibiotic exposure rate (Φ) /hab/day	0.007	0.002	Guillemot et al.[9]
**Data from the clinical studies used for model calibration**			
Initial carriage prevalence	0.64	0.47	Amoxicillin study[1] Erythromycin *study*[3]
Initial resistance proportion	0.19	0.53	
After treatment carriage prevalence	0.13	0.25	
After treatment resistance proportion	0.67	0.91	
Duration of treatment ($1/\gamma$) (days)	7	8	
**Calibrated parameters on antibiotic-induced decolonization**			
Rate of antibiotic-induced decolonization rate on susceptible strains	$\delta_{PSSP}^{Amox} = 1$	$\delta_{S}^{Macro} = 1$	Calibrated
Rate of antibiotic-induced decolonization rate on resistant strains	$\delta_{PNSP}^{Amox} = 0.090$	$\delta_{R}^{Macro} = 0.035$	

Supplementary Table.1 Characteristics of studies used to calibrate antibiotic-induced decolonization rates, model parameters used for calibration, and resulting calibrated decolonization rates.

Parameter	Interpretation	Unit	Value		Sources	
			French analysis	European analysis	French analysis	European analysis
**Parameters for the <5y groups**						
$\boldsymbol{PNSP^{init}}$	Initial *S. pneumoniae* penicillin-non-susceptible proportion in carriers	-	0.43	See table. 3	Pneumococcal National Reference Centre (CNRP) 2022 activity report [10]	European Antimicrobial Resistance Surveillance Network (EARS-Net)[6]
$\boldsymbol{MR^{init}}$	Initial *S. pneumoniae* macrolide-resistant proportion in carriers	-	0.28	See table. 3		
$\boldsymbol{Rcr^{init}}$	Initial proportion of macrolide-resistant strains among penicillin-non-susceptible strains	-	0.58	See table. 3		
$\boldsymbol{N}$	Population size	-	3 850 000		French National Institute of Statistics and Economical Studies (INSEE)[11]	
$\boldsymbol{\gamma^{Amox}}$	Rate of return to antibiotic unexposed compartment (1/duration of amoxicillin treatment	days^{-1}	1/7		French Health Authority (HAS)[12]	
$\boldsymbol{\gamma^{Macro}}$	Rate of return to antibiotic unexposed compartment (1/duration of macrolide treatment)	days^{-1}	1/7			
$\boldsymbol{\Phi^{Amox}}$	Beta-lactam exposure rates	year^{-1}	0.95	See table. 3	Geodata in public health (Géodes)[13]	European Surveillance of Antimicrobial Consumption Network (ESAC-Net)[5]
$\boldsymbol{\Phi^{Macro}}$	Macrolide exposure rates	year^{-1}	0.083	See table. 3		
$\mathbf{p^{init}}$	Carriage prevalence		0.52		Literature[15,16]	
$\boldsymbol{1/\lambda}$	Duration of colonization	days	43		Literature[7,8]	
$\boldsymbol{\beta}$	Transmissibility rate	days^{-1}	0.056	Calibrated	Calibrated	
$\boldsymbol{\varepsilon_{IPD}}$	Pneumococcal invasion rate	days^{-1}	4.8×10^{-7}		Epibac[17]	
$\boldsymbol{\theta}$	Replacement penalty		0.5		Assumed	
**Parameters by levels of resistance (k),** $\boldsymbol{if\ Antibiotic = Amoxicilline,\ k \in \{MIC1, MIC2, \dots, MIC8\}\ if\ Antibiotic = Macrolide, k \in \{S, R\}}$						
$\boldsymbol{d_k^{Amox}}$	Distribution of S.p penicillin susceptibility levels according to the MIC	-	[0.47,0.075, 0.075, 0.085, 0.09, 0.12, 0.075, 0.01]		Pneumococcal National Reference Centre (CNRP) 2022 activity report [10]	
$\boldsymbol{\delta_k^{Amox}}$	Amoxicillin-induced decolonization rate	days^{-1}	[1,0.93,0.47,0.06, 0, 0,0,0]		Literature[1,4] and calibrated	
$\boldsymbol{f_k^{Amox}}$	Penicillin fitness cost	-	[1, 0.999, 0.992, 0.942, 0.919, 0.919, 0.919, 0.919]		Calibrated	
$\boldsymbol{a_k^{Amox}}$	Rate of transmission under amoxicillin exposure	-	[0, 0, 0, 1, 1, 1, 1, 1]		Assumed	
$\boldsymbol{\delta_k^{Macro}}$	Macrolide-induced decolonization rate	days^{-1}	[1, 0.035]		Calibrated	
$\boldsymbol{f_k^{Macro}}$	Macrolide fitness cost	-	[1, 0.995]		Calibrated	
$\boldsymbol{a_k^{Macro}}$	Rate of transmission under macrolide exposure	-	[0, 1]		Assumed	

Supplementary Table.2 Parameters of the model in a French and European context

Country	$PNSP^{init}$	MR^{init}	MDR^{init}	Rcr^{init}	Φ^{Amox}	Φ^{Macro}
**Austria**	0,052	0,143	0,48	0,02	0,114	0,060
**Belgium**	0,18	0,165	0,54	0,10	0,248	0,114
**Croatia**	0,183	0,228	0,86	0,16	0,227	0,131
**Czechia**	0,057	0,105	0,61	0,04	0,125	0,109
**Denmark**	0,096	0,051	0,31	0,03	0,272	0,050
**Estonia**	0,046	0,061	0,89	0,04	0,106	0,075
**Finland**	0,146	0,133	0,59	0,09	0,098	0,017
**France**	0,32	0,23	0,63	0,20	0,365	0,102
**Germany**	0,078	0,066	0,28	0,02	0,094	0,050
**Hungary**	0,124	0,14	0,51	0,06	0,109	0,125
**Ireland**	0,196	0,126	0,38	0,08	0,239	0,144
**Italy**	0,1	0,24	0,65	0,07	0,236	0,205
**Lithuania**	0,083	0,183	0,55	0,05	0,180	0,098
**Netherlands**	0,062	0,033	0,15	0,01	0,080	0,061
**Norway**	0,061	0,054	0,54	0,03	0,161	0,025
**Poland**	0,188	0,291	0,79	0,15	0,173	0,158
**Portugal**	0,144	0,191	0,68	0,10	0,218	0,082
**Slovenia**	0,064	0,07	0,33	0,02	0,151	0,054
**Spain**	0,223	0,274	0,59	0,13	0,328	0,089
**Sweden**	0,075	0,048	0,35	0,03	0,146	0,020

Supplementary Table.3 Country-specific pharmaco-epidemiological parameters in the average population

Data on pneumococcal resistance were sourced from the European Antimicrobial Resistance Surveillance Network (EARS-Net)[6], using 2021 data. Data on beta-lactam and macrolide consumption were obtained through the European Surveillance of Antimicrobial Consumption Network (ESAC-Net)[5], using 2021 data.

III. Descriptions

Supplementary Description.1 Absolute variation in *S. pneumoniae* invasive disease incidence, considering a 1-year 50% beta-lactam shortage in the French context, with 3.8 million children under 5 years of age.

Applied to France, and considering the 3.8 million children under 5 years of age, we estimate that a 50% shortage over a year could lead, in case of reduction of beta-lactam consumption frequency (strategy S1), to an increase in overall infections by 12 [10,14] IPD cases. This includes a decrease in resistant infections of -10 [-5,-13] $\mathrm{IPD_{PNSP}}$ and -5 [-2,-6] $\mathrm{IPD_{MRSP}}$ cases and a larger increase in susceptible infections of 21 [17,23] $\mathrm{IPD_{SSP}}$ cases. Reducing beta-lactam treatment duration (strategy S2) or prescribed daily dose (strategy S3) could also increase overall infections (by 5 [5,6] and 4 [4,5] IPD cases, respectively), with S2 leading to a decrease in resistant infections (-4 [-2,-6] $\mathrm{IPD_{PNSP}}$ and -2 [-1,-3] $\mathrm{IPD_{MRSP}}$ cases), and S3 leading to an increase in resistant infections (13 [9,15] $\mathrm{IPD_{PNSP}}$ and 7 [5,8] $\mathrm{IPD_{MRSP}}$ additional cases). Finally, switching from beta-lactam to macrolide prescription in case of shortage (strategy S4) does not affect the overall IPD incidence, and results in shifting resistance patterns, with 7 [5,10] $\mathrm{IPD_{MRSP}}$ and 5 [3,6] $\mathrm{IPD_{MDRSP}}$ additional cases but a decrease of -5 [-3,-6] $\mathrm{IPD_{PNSP}}$ cases.

Supplementary Description.2 Calculation explanation of the Number of DDD

The calculation is based on the generic formula: Number of DDD/1,000/day = (Volume of active substance sold × 1,000 inhabitants) / (DDD × study population).

In the case of amoxicillin DDD_{Amox}=1.5 (according to the WHO for a 1000mg tablet) and in the event of a 50% shortage

- For strategy S1: the number of individuals exposed is divided by 2:

$$NumberOfDDD/1{,}000_{Amox} = \frac{(NbExposedIndividual_{Amox}/2) * NbIntake * Dose * 1{,}000}{DDD_{Amox} * PopulationSize}$$

- For strategy S3: the administered dose is reduced from 1g to 0.5g, so the dose is divided by 2.

$$NumberOfDDD/1{,}000_{Amox} = \frac{NbExposedIndividual_{Amox} * NbIntake * (Dose/2) * 1{,}000}{DDD_{Amox} * PopulationSize}$$

- For strategy S2: the duration of beta-lactam treatment is halved, which consequently reduces the exposure prevalence by half.

$NbIntake$= number of daily intakes (3 times daily for amoxicillin)

Since both strategies correspond to the same reduction in total active substance consumed, the total number of amoxicillin DDD/1,000 children/day is identical across S1, S2, and S3.

IV. Equations of the model

$$\frac{dUS^i}{dt} = \mu_0 + \lambda_i \sum_{l=1}^{8}\sum_{n=S}^{R}\left(UC_{CMIl\_n}{}^{i}\right) + \gamma^{Amox}AES^i + \gamma^{Macro}MES^i - \left(\mu_i + \varphi_i^{Amox} + \varphi_i^{Macro}\right)US^i$$
$$- \sum_{j=1}^{4}\sum_{l=1}^{8}\sum_{n=S}^{R} v_i C_{ij}\left(\frac{f_l \cdot f_m\left(UC_{CMIl\_n}{}^{j} + AEC_{CMIl\_n}{}^{j} + MEC_{CMIl\_n}{}^{j}\right)}{N}\right)US^i$$

$$\frac{dUC_{CMIk\_m}{}^{i}}{dt} = \gamma^{Amox}AEC_{CMIk\_m}{}^{i} + \gamma^{Macro}MEC_{CMIk\_m}{}^{i} - \left(\mu_i + \varphi_i^{Amox} + \varphi_i^{Macro} + \lambda_i\right)UC_{CMIk\_m}{}^{i}$$
$$+ \sum_{j=1}^{4} v_i C_{ij}\left(\frac{f_k \cdot f_S\left(UC_{CMIk\_m}{}^{j} + AEC_{CMIk\_m}{}^{j} + AEC_{CMIk\_m}{}^{j}\right)}{N_j}\right)US^i$$
$$+ \theta \sum_{j=1}^{4} v_i C_{ij}\left(\frac{f_k \cdot f_S\left(UC_{CMIk\_m}{}^{j} + AEC_{CMIk\_m}{}^{j} + AEC_{CMIk\_m}{}^{j}\right)}{N_j}\right)\sum_{l=1}^{8}\sum_{n=S}^{R;\, l\_n \neq k\_m} UC_{CMIl\_n}{}^{i}$$
$$- \theta \sum_{j=1}^{4}\sum_{l=1}^{8}\sum_{n=S}^{R;\, l\_n \neq k\_m} v_i C_{ij}\left(\frac{f_l \cdot f_m\left(UC_{CMIl\_n}{}^{j} + AEC_{CMIl\_n}{}^{j} + MEC_{CMIl\_n}{}^{j}\right)}{N}\right)UC_{CMIk\_m}{}^{i}$$

$$\frac{dAES^i}{dt} = \sum_{l=1}^{9}\sum_{n=S}^{R}\left(\lambda_i + \delta_l^{Amox}\right)AEC_{CMIl\_n}{}^{i} + \varphi_i^{Amox}US^i - \left(\mu_i + \gamma^{Amox}\right)AES^i$$
$$- \sum_{j=1}^{4}\sum_{l=1}^{8}\sum_{n=S}^{R} a_l v_i C_{ij}\left(\frac{f_l \cdot f_m\left(UC_{CMIl\_n}{}^{j} + AEC_{CMIl\_n}{}^{j} + MEC_{CMIl\_n}{}^{j}\right)}{N}\right)AES^i$$

$$\frac{dAEC_{CMIk\_m}{}^{i}}{dt} = \varphi_i^{Amox}UC_{CMIk\_m}{}^{i} - \left(\mu_i + \gamma^{Amox} + \lambda_i + \delta_k^{Amox}\right)AEC_{CMIk\_m}{}^{i} + \sum_{j=1}^{4} a_k v_i C_{ij}\left(\frac{f_k \cdot f_S\left(UC_{CMIk\_m}{}^{j} + AEC_{CMIk\_m}{}^{j} + AEC_{CMIk\_m}{}^{j}\right)}{N_j}\right)AES^i$$
$$+ \theta \sum_{j=1}^{4} a_k v_i C_{ij}\left(\frac{f_k \cdot f_S\left(UC_{CMIk\_m}{}^{j} + AEC_{CMIk\_m}{}^{j} + AEC_{CMIk\_m}{}^{j}\right)}{N_j}\right)\sum_{l=1}^{8}\sum_{n=S}^{R;\, l\_n \neq k\_m} AEC_{CMIl\_n}{}^{i}$$
$$- \theta \sum_{j=1}^{4}\sum_{l=1}^{8}\sum_{n=S}^{R;\, l\_n \neq k\_m} a_l v_i C_{ij}\left(\frac{f_l \cdot f_m\left(UC_{CMIl\_n}{}^{j} + AEC_{CMIl\_n}{}^{j} + MEC_{CMIl\_n}{}^{j}\right)}{N}\right)AEC_{CMIk\_m}{}^{i}$$

$$\frac{dMES^i}{dt} = \sum_{l=1}^{9}\sum_{n=S}^{R}\left(\lambda_i + \delta_n^{Macro}\right)MEC_{CMIl\_n}{}^{i} + \varphi_i^{Macro}US^i - \left(\mu_i + \gamma^{Macro}\right)MES^i$$
$$- \sum_{j=1}^{4}\sum_{l=1}^{8}\sum_{n=S}^{R} a_m v_i C_{ij}\left(\frac{f_l \cdot f_m\left(UC_{CMIl\_n}{}^{j} + AEC_{CMIl\_n}{}^{j} + MEC_{CMIl\_n}{}^{j}\right)}{N}\right)MES^i$$

$$\frac{dMEC_{CMIk\_m}{}^{i}}{dt} = \varphi_i^{Macro}UC_{CMIk\_m}{}^{i} - \left(\mu_i + \gamma^{Amox} + \lambda_i + \delta_m^{Macro}\right)MEC_{CMIk\_m}{}^{i} + \sum_{j=1}^{4} a_S v_i C_{ij}\left(\frac{f_k \cdot f_S\left(UC_{CMIk\_m}{}^{j} + AEC_{CMIk\_m}{}^{j} + AEC_{CMIk\_m}{}^{j}\right)}{N_j}\right)MES^i$$
$$+ \theta \sum_{j=1}^{4} a_S v_i C_{ij}\left(\frac{f_k \cdot f_S\left(UC_{CMIk\_m}{}^{j} + AEC_{CMIk\_m}{}^{j} + AEC_{CMIk\_m}{}^{j}\right)}{N_j}\right)\sum_{l=1}^{8}\sum_{n=S}^{R;\, l\_n \neq k\_m} MEC_{CMIl\_n}{}^{i}$$
$$- \theta \sum_{j=1}^{4}\sum_{l=1}^{8}\sum_{n=S}^{R;\, l\_n \neq k\_m} a_m v_i C_{ij}\left(\frac{f_l \cdot f_m\left(UC_{CMIl\_n}{}^{j} + AEC_{CMIl\_n}{}^{j} + MEC_{CMIl\_n}{}^{j}\right)}{N}\right)MEC_{CMIk\_m}{}^{i}$$

Supplementary Equation.1 Transmission model equations of pneumococcus colonisation

Supplementary References

1. Lewnard, J. A. *et al.* Impact of Antimicrobial Treatment for Acute Otitis Media on Carriage Dynamics of Penicillin-Susceptible and Penicillin-Nonsusceptible Streptococcus pneumoniae. *The Journal of Infectious Diseases* **218**, 1356–1366 (2018).
2. Byrd, R. H., Lu, P., Nocedal, J. & Zhu, C. A Limited Memory Algorithm for Bound Constrained Optimization. *SIAM J. Sci. Comput.* **16**, 1190–1208 (1995).
3. Varon, E. *et al.* Impact of Antimicrobial Therapy on Nasopharyngeal Carriage of Streptococcus pneumoniae, Haemophilus influenzae, and Branhamella catarrhalis in Children with Respiratory Tract Infections. *Clinical Infectious Diseases* **31**, 477–481 (2000).
4. de Velde, F. *et al.* Non-linear absorption pharmacokinetics of amoxicillin: consequences for dosing regimens and clinical breakpoints. *Journal of Antimicrobial Chemotherapy* **71**, 2909–2917 (2016).
5. AMC | European Centre for Disease Prevention and Control. https://qap.ecdc.europa.eu/public/extensions/AMC2_Dashboard/AMC2_Dashboard.html#eu-consumption-tab.
6. European Centre for Disease Prevention and Control. & World Health Organization. *Antimicrobial Resistance Surveillance in Europe 2023: 2021 Data.* (Publications Office, LU, 2023).
7. Högberg, L. *et al.* Age- and Serogroup-Related Differences in Observed Durations of Nasopharyngeal Carriage of Penicillin-Resistant Pneumococci. *Journal of Clinical Microbiology* **45**, 948–952 (2007).
8. Ekdahl, K. *et al.* Duration of Nasopharyngeal Carriage of Penicillin-Resistant Streptococcus pneumoniae: Experiences from the South Swedish Pneumococcal Intervention Project. *Clinical Infectious Diseases* **25**, 1113–1117 (1997).

9. Guillemot, D. *et al.* Reduction of Antibiotic Use in the Community Reduces the Rate of Colonization with Penicillin G—Nonsusceptible Streptococcus pneumoniae. *Clin Infect Dis* **41**, 930–938 (2005).
10. Pneumococcal National Reference Centre. 2022 Activity Report. (2023).
11. Insee. Population projections 2021-2070. https://www.insee.fr/fr/statistiques/5894083?sommaire=5760764.
12. Haute Autorité de Santé. Recommended choice and duration of antibiotic therapy for common bacterial infections. https://www.has-sante.fr/jcms/p_3278764/fr/choix-et-durees-d-antibiotherapie-preconisees-dans-les-infections-bacteriennes-courantes (2021).
13. Géodes - Santé publique France - Indicators: maps, data and graphics-Number of prescriptions of beta-lactam antibiotics (ATC: J01C) in general practices - under 5 years old. https://geodes.santepubliquefrance.fr/#c=indicator&f=04&i=antibio_conso.nb_pres_j01c&s=2023&t=a02&view=map2.
14. Géodes - Santé publique France - Indicators: maps, data and graphics-Number of prescriptions in towns for macrolides (ATC: J01F) - under 5 years old. https://geodes.santepubliquefrance.fr/#c=indicator&f=04&i=antibio_conso.nb_pres_j01f&s=2023&t=a02&view=map2.
15. Southern, J. *et al.* Pneumococcal carriage in children and their household contacts six years after introduction of the 13-valent pneumococcal conjugate vaccine in England. *PLOS ONE* **13**, e0195799 (2018).
16. Sanchez Picot, V. *et al.* Epidemiology and serotype distribution of Streptococcus pneumoniae carriage among influenza-like illness cases in metropolitan Vientiane, Lao PDR: a community-based cohort study. *Front Public Health* **11**, 1124016 (2023).
17. Santé publique France. EPIBAC public health bulletin: surveillance of invasive bacterial infections in 2021. https://www.santepubliquefrance.fr/maladies-et-traumatismes/maladies-et-infections-respiratoires/infections-a-

pneumocoque/documents/bulletin-national/bulletin-de-sante-publique-epibac-surveillance-des-infections-invasives-bacteriennes-en-2021 (2022).
