# Supplementary material for: Modelling impacts of paediatric amoxicillin shortage management on pneumococcal resistance and invasive disease in Europe
Source: Nat Commun. 2026 May 19;17:6592. doi: 10.1038/s41467-026-72777-y (PMC13381528; doi:10.1038/s41467-026-72777-y)
Supplement: Supplementary file 3 — Transparent Peer Review file [file 41467_2026_72777_MOESM3_ESM.pdf]

nature portfolio

Peer Review File

Modelling impacts of paediatric amoxicillin shortage management on pneumococcal resistance and invasive disease in Europe

Corresponding Author: Ms Aurélie MAURIN

This file contains all reviewer reports in order by version, followed by all author rebuttals in order by version.

Version 0:

Reviewer comments:

Reviewer #1

(Remarks to the Author)
Nature Comms Review

Maurin et al.,
Esmita Charani:
This is a good paper that is well written. It is positive that there is attention on the issue of shortages. There are many difficulties in collecting real life impact data of antibiotic shortages and the use of modelling is appropriate in the context. There has been a Europe wide shortage of amoxicillin over the last couple of years and it would have been useful to bring in some of that real-time data too. The disclaimer for conducting this review is that I do not have mathematical modeling expertise. Hence I reviewed this from a pragmatic clinical and practice perspective.
My comments below are in order of appearance in manuscript and (for expediency) not in order of importance.
Abstract
The link to bacterial consequences are hypothesized but not backed by evidence yet.
Introduction
Generally well-written and brings in the existing body of literature on antibiotic shortages. Lines 75-79 The existing literature, mainly from high income settings and hospitals describes a limited number of studies that have data on impact of shortages on infection and points to a mixed data. What can be said is that the increasing occurrence of shortages of Access and Reserve category agents is compromising optimized antibiotic care and causing knock on shortages of substitute agents. It also depletes existing antibiotic stewardship programmes where they exist.

Lines 80 to 87 The impact of shortages on any given infection or resistance epidemiology is not well understood, it is not specific to pneumococcal disease. Did the dynamic modelling work on a hypothetical shortage scenario or was it developed using the existing global shortage of amoxicillin?

Results/methods
In a real case scenario reducing dose would probably be more relevant if there were substandard formulations.
For shortages the most common option for pediatric medicine is switching to a different agent, using adult formulations and crushing/administering.
Line 566 – error message for figure needs to be corrected
Discussion
Line 421 The impact of shortages also depend on stewardship capabilities, healthcare infrastructure, procurement and responsiveness of the health system to dealing with the shortage. So to develop a model that purely considers the dosage and switching is a limitation.
The model works for high income countries with universal health coverage and assumed otherwise good access to healthcare and antibiotics. The model would be very different for low-resource settings where access to medicines, including antibiotics, can be via informal routes, with higher rates of exposure to substandard and falsified medicines, and paediatric populations with different vulnerability and epidemiological (and vaccination) profile for pneumococcal diseases.
The capability of stockpiling, contractual pricing also influences the response to dealing with shortages. So on lines 555 it would be very helpful to identify what the authors mean by ‘establishing clear public health priorities’ bearing in mind this

needs to be contextualized.
Stewardship only features in the discussion and then in concluding paragraph. Could the authors have considered building in the consequence of effective stewardship into the model? Existing evidence shows that managing antibiotic shortages whilst benefiting from stewardship can also compromise it leading to knock on effect on quality of antibiotic use in health systems. So the ecological and epidemiological consequences can be beyond a single pathogen or diseases/population.

Minor suggestions and typos
Can remove 'both' from line 27
Line 62 change amoxicillin forms to formulations
Line 75 should be changed to USA
Throughout replace multivariate with multivariable
Ritika Kondal:
General Comments: Well written. Interesting and meticulously conducted study. Model development was done with consideration of actual scenarios like, resistance, fitness cost and competition parameters. This gives an edge to the complete mathematical modelling study which is not backed by actual data.
Introduction:
Line 79: One line about how shortages can impact carriage prevalence can be added. It will give context to the model researchers have developed, as they have taken these parameters in model development.
Line 85: Can be written different prescribing strategies instead of "patterns" as through the paper, authors have used the term strategies

Methods:
Line 569: After looking at the colonization status, described later and in the results also, this line is contradictory.
If exposed children will always have resistant strains exposure, then why to mention susceptible strains?
Line 620: Steady state of which index/ parameter was assumed?
Line 624: full form of PNSP needs to be given before
Meaning of gamma and lambda is not explained in the methods section. It's explained in figures legends. But needs to mentioned in the method section for better clarity.
Line 664: Are authors want to say about rate of infection in child? The sentence seems incomplete.
Line 675- 678: The sentence should come before in the method section for better clarity.
Results:
Fig 2: Colour of the figure needs to be corrected. (grey scale is not visible in the figure)

Line 161-164: How could strategy 1-3 will have an effect on macrolide exposure? Not able to understand.

Discussion:
Line 519-527: Its repetition of the results from Fig 6. Possible explanation for the same could be given.
This study outcomes can predict the impact of different strategies in HIC countries where a defined system and protocol is followed for management of infection. However, when it comes LMIC's due to high burden of infection, most of the primary and secondary care facilities do not have the resistance mapping labs. So, variability of proportion of resistance strains cannot be applied to LMIC settings. Authors should mention the applicability of the study in HIC.

(Remarks on code availability)

Reviewer #2

(Remarks to the Author)
The manuscript fills a gap in understanding the impact of policies with this natural occurring experiment. The authors elicit a relevant set of policy options and outcomes and those from the data accrued. This study also sheds some understanding on the nuances and implications when we think of reducing antibiotic prescribing, and its impact on antimicrobial resistance and infection burden. The authors considered alternative endpoints to illustrate the impact of interventions on different realms of policy.

The quality of the data is good and suited to be used in a simulation for European countries. This is also only possible given the quality of ECDC data. The sensitivity analysis shows that main drivers of outcomes are considered in the analysis and interventions seem to have a linear and expected effect on outcomes.

Some of the findings speak to the broader discussion of appropriate use and prescription of antibiotics in other regions. However, there is very limited data from those and also extreme scenarios that make non-linearities more likely to emerge. These caveats could perhaps be considered in a couple of phrases in lines 537 onwards.

I think the authors do a fair assessment of the extent to which their conclusions would be valid and can inform decision making, also leaving room for the normative assessment of policy aim ranking. I also believe the author's findings speak to the broader discussion of appropriate use of antibiotics. This highlights that stewardship capacity could make use of associated interventions that mitigates these undesirable effects and optimises prescribing.

Suggested improvements

1.It could be useful for some readers to divide figure 6 into high-vs-low prescriber groups. It could make it easier to glance trends that are spelled in the text. It would also make some comparisons interesting, as I imagine Denmark being in the low-prescribing group yet exhibits some trends similar to high-prescribers.

2.Please specify the “initial prevalence” on line 509.

3.Lines 498-511 mention the limitations of using the French data and how the sensitivity analysis showed that the greatest impact is accounted for in the simulations. However, it does not mention the fact that France was regarded as a high-prescriber yet used to draw conclusions on low-prescriber countries.

4.Lines 537-545 rightly highlight the implications of this study for stewardship interventions. This highlights the importance of associated intervention that mitigates the initial effects from stewardship which could be unpopular.

5.Typo on line 158 : “and per day”

6.Typo on line 202: “ all S. pneumoniae”

7.Line 566 on materials and methods, model description, the reference to figure 1 shows an error.

8.Line 625 on materials and methods, model description, reference to supplementary methods shows an error.

(Remarks on code availability)

Reviewer #3

(Remarks to the Author)
I co-reviewed this manuscript with one of the reviewers who provided the listed reports. This is part of the Nature Communications initiative to facilitate training in peer review and to provide appropriate recognition for Early Career Researchers who co-review manuscripts.

(Remarks on code availability)

Reviewer #4

(Remarks to the Author)
Title: Impact of amoxicillin shortage on pneumococcal resistance and IPD in children: evaluation of different management strategies in European countries

This study by Maurin et al develops a novel varying resistance level model of beta-lactam and macrolide resistance in S. pneumoniae. They explore a highly pertinent question around the impact of antibiotic shortages and develop multiple interesting results that will be useful to policy makers and in considering the future impacts of these increasingly frequent events. I have some points of clarification and a few recommendations in terms of putting the results in context but otherwise believe that this a solid piece of work. My only major concern is around understanding exactly what was done in terms of model fitting but I hope this is only a clarification issue.

Major
- Should more be made of the impact on MDR-strain increases? What are the treatment options for these infections? Surely this dual resistance is the most worrying compared to the mono-resistances and is the one to focus on compared to IPD increase?
- Throughout care needs to be taken that fitness cost and relative fitness are not used incorrectly. I think that a “fitness cost of 0.995” for example should be a “relative fitness of 0.995”. Also Table 1 should be relative fitness not cost?
- The calibration section is unclear – paragraph starting line 608. What is the likelihood / L-BFGS algorithm / initial conditions? why only after 1 year do you do the fitting? normal distribution of what? I don’t understand how this matches then what is done in the next paragraph? How can you fit the transmission / fitness cost without antibiotic exposure? Line 622 – what change? Why is least squares now being used no the L-BFGS?
- More needs to be included in the introduction and discussion on the beta-lactam / macrolide / IPD treatment balance. The introduction needs more on macrolide use and resistance. Isn’t MDR the biggest issue as then there are no (?) treatment options? What is the difference between beta-lactams and macrolides in terms of cost / efficacy / mortality / AWaRE classifications?
- The writing is sloppy in places – a grammar check needs to be done throughout – e.g. line 114 “a” is missing before rate, line 599 “despite colonized” needs correcting. The discussion especially needs a careful read through. Similarly there are some “Error missing source” etc.
Minor

Abstract: I think more needs to be included in here about the main analysis which is based on the French situation with less focus on the country comparisons (only one figure on this and it uses many French parameters).
Abstract: I think "consequences" needs to be clearer – add into previous paragraph?
Line 78: Ref 15 says in abstract "Multivariable analyses identified significant associations of ABR with country-level selecting antibiotic sales, but only in fluoroquinolone-resistant-Escherichia coli, fluoroquinolone-resistant-Pseudomonas aeruginosa, and carbapenem-resistant-A baumannii." so not for S. pneumoniae?

Introduction: overall couldn't shortages be a good think in terms of reducing overall resistance? Line 76: I don't understand the link to resistance here.

Introduction: Please include more on how policymakers / prescribers could or might be influenced by modelling results showing the balance between resistance and disease. Who makes decisions following shortages on how it affect use? Either here or in the discussion.

Introduction: I think this also could do with a sentence or two about the novelty of the MIC variation included in the model – setting up that S. pneumoniae has variation in this and the importance for resistance transmission / antibiotic exposure impact. Moreover why this variation is more for beta-lactams than macrolides.

Results

Figure 1: what are lambda / phi / delta? Either include legend or refer to table which describes them
Figure 1: k is numeric I think so it should be k is a member of {1,2,..,8} not CM1 etc

Line 110: evidence for a fitness cost? And its potential size?
Line 110: any evidence re: the difference in exposure? Is this ok?
Line 113: does this protection wane? Otherwise seems a strong assumption?
Line 114: based on descriptions elsewhere, can you clarify here that you don't actually model this infection but you calculate a fixed number of IPDs based on the number of colonized individuals?
Figure 2: why do s1 and s2 have such similar impacts? And why doesn't S3 reduce the DDD more than s1 / s2 ?
Line 125: Could you specify / clarify that you refer / use pristinamycin as your example macrolide for the DDD calculation? And add to your methods. Is this commonly used in France? And an OK example macrolide?
Line 141: Should uncertainty in fitness costs be explored? I think it wasn't but could you comment in the discussion on how sure you are in your estimates of these? Variation here would have a big impact on the 1 yrs timings but I don't believe this is in your PRCC as it was a fitted parameter.
Line 151: I struggle to understand this in terms of what this means for beta-lactam and macrolide use and then for Figure 3. Please clarify.
Line 155: Comparing between the strategies would be useful results to include here I think.
Line 165: in this paragraph could add something that all though some substantial percentage reductions the absolute levels don't change enough to affect empiric prescribing decisions. Indeed the differences between the levels of resistance is quite minimal.
Line 216: I don't understand why the linear trend is broken? Please discuss the underlying mechanism more here or in the discussion.
Line 257: is this result discussed / explored in the discussion?
Figure 5: please emphasise (add thick line or arrow?) which of these initial conditions is the "France" that you model in previous results.
Line 335: please make clear which parameters are "French" and which vary in this analysis
Figure 6: I don't understand what the numbers in this figure are. Variation over what? Is it just the "reduction" vs pre shortage 1 year earlier?
Line 347: between countries "and strategies" ?
Methods
Thank you for sharing the code.
Line 636: where does the 52% come from? And is "optimal" the correct word to use here and for the fitness cost values?
Line 638: what is "pneumococcus in otitis" and how did you use this data? This is linked to the major issue with not understanding your calibration methods?
Line 663: Could you emphasise in this paragraph which is the one used in the main results? I think 50% reduction?
Line 668: can you explain by how much S3 reduces the rate?
Line 690: where are these reviewed studies from? France? How much variation in location might explain the variation seen?
Line 692: how did you define a "peak"?
Line 694: does iteration number mean the number of samples? Perhaps change to that if so, or explain what iteration number means for here and line 709.
Line 696: was this just for France?
Line 722: why only use 2021 data? Is there a trend in resistance / infection incidence before after? Is this ok in terms of COVID-19 impacts?
Line 728: does "tested" here mean those with drug susceptibility tests?

Discussion
- please add in something about how the changes in resistance prevalence are relatively minor (e.g. Figure 2b) and probably wouldn't affect empiric prescribing choices. As in resistance below 10% or above 20% wouldn't change what is prescribed or perhaps it would in France?

- line 391: do they really “vary widely”? I would context the “widely”.
- line 400: can you mention the strategy names in this paragraph to link easily to the results? And expand acronyms on first use?
- line 400: can you add in a discussion of S3 too in this paragraph?
- line 443: please expand on the lack of inclusion of emergence of resistance – is this ok for both beta-lactam and macrolide resistance? What are the mechanisms of that? How does this potentially bias your results? I’m thinking here about the differential impact of the strategies – fewer doses vs higher DDD may have different impacts on emergence.
- line 494: could IPD even have been reduced / not affect with the right antibiotic usage?
- line 512: could you comment on which strategies are potentially implementable? And how they would be enforced? Is it likely that policymakers could recommend one strategy or would a combination be the most likely to happen?
- line 519: This paragraph needs a “so what?”. Is 4 ok? is this within annual fluctuation? how many IPD lead to death etc? Any decisions made on this in the past?
- line 536: linked to the major comments, I think here more needs to be made on amoxicillin resistance vs macrolide resistance vs dual resistance and which is more concerning and why.
- line 540: up to s4?
- line 555: Can you make any conclusions on whether antibiotic shortages are overall bad? and in what way? As in, could they in fact reduce antibiotic resistance? Can you comment on the overall likely effect.

(Remarks on code availability)

Version 1:

Reviewer comments:

Reviewer #2

(Remarks to the Author)
Thank you for addressing all comments and questions from reviewers.

(Remarks on code availability)
The code is sufficiently documented

Reviewer #3

(Remarks to the Author)
I co-reviewed this manuscript with one of the reviewers who provided the listed reports. This is part of the Nature Communications initiative to facilitate training in peer review and to provide appropriate recognition for Early Career Researchers who co-review manuscripts.

(Remarks on code availability)
Not applicable

Reviewer #4

(Remarks to the Author)
Thank you for these revisions. They are mostly highly appropriate and I think have strengthened the manuscript. However I have two remaining issues.

Firstly, a minor point that care still needs to be taken on the fitness cost - line 737 this is relative fitness not cost?

Secondly, the calibration is still unclear (line 776 onwards). This just needs a few minor tweaks.
- How were beta and f_k “calibrated”? by hand?
- Which of the parameters in Supplementary table 1 did you “fit to”? I think it is the “after treatment” carriage and resistant proportion but this is not totally clear. Which of the parameters in this table are you able to include in your model (e.g. population size? Age?) and which are parameters from the data there just for information? This in unclear between line 800 and then 802 where it says that you use levels from “before antibiotic exposure”?
- Supplementary table 1: what is “calibrated data”? do you mean “estimated parameters”?
- I would suggest that you split supplementary Table 1 into
o Study information
o Data levels fitted to or something
o Fitted parameters (not data)
- What is the objective function? (line 798)
- Line 818: I am still not 100% sure what the observed values were – overall proportion of resistant strains with the 8 MIC values in Table 1? Could you just link the sentences in 818-822 more clearly so that the “eight” clearly links to the “distribution of MIC values” perhaps linked to those shown in Table 1 and that these levels are the observed values?
- Line 830 – how was the optim function used in the two step first process? As mentioned above was the calibration partly by

hand?

It is great to see the new sensitivity analysis and thanks for pointing out the importance of relative not absolute in your results.

Unfortunately I wasn't able to reproduce your results with the existing code - please provide more structure and check the dependencies.

(Remarks on code availability)
Although some packages are set up initially, the internal code needed many more packages (e.g. rayshadr, triangle)

And then this file was missing or rather the setup pointed to something on the author's computer but I don't have it: gen <- odin::odin("C:\\Users\\maurinau\\OneDrive - LECNAM\\Stage\\StagePasteur\\codeRStage\\DeuxAgeDeuxResistanceOdin.R")

The authors need to provide more structured guidance on the order and way to run the R / Rmd file and check the file address dependencies.

I could not reproduce the work in the current state - using the here package and giving me more instructions on how to use the scripts would enable this. A lot of the code is here which is great but more commenting and support is needed.

Version 2:

Reviewer comments:

Reviewer #4

(Remarks to the Author)
Thank you - comprehensive response, nothing else from me on the text.

(Remarks on code availability)
I cannot run the code - I get multiple errors.
Initially in the first RMd I think line 102 should not be "Optim" but Optimi with an i?
The first two files then fun but then I cannot run the UndertaintyAnalysisFigure1 as I do not have "rayshader" - its not in the list of needed installations at the start and then, when I try to install from "install.packages" it fails and I get "OpenGL is not available in this build" etc.

Please double check with a clean computer set up that this all works. I'm happy to try this again once you have had another look. Please also include headings and descriptions of what each file does.

Open Access This Peer Review File is licensed under a Creative Commons Attribution 4.0 International License, which

permits use, sharing, adaptation, distribution and reproduction in any medium or format, as long as you give appropriate credit to the original author(s) and the source, provide a link to the Creative Commons license, and indicate if changes were made.
In cases where reviewers are anonymous, credit should be given to 'Anonymous Referee' and the source.
The images or other third party material in this Peer Review File are included in the article's Creative Commons license, unless indicated otherwise in a credit line to the material. If material is not included in the article's Creative Commons license and your intended use is not permitted by statutory regulation or exceeds the permitted use, you will need to obtain permission directly from the copyright holder.
To view a copy of this license, visit https://creativecommons.org/licenses/by/4.0/

Response to reviewers' comments :

Throughout this document, line numbers refer to the revised version of the manuscript (without track changes).

Reviewer #1

Esmita Charani:

This is a good paper that is well written. It is positive that there is attention on the issue of shortages. There are many difficulties in collecting real life impact data of antibiotic shortages and the use of modelling is appropriate in the context. There has been a Europe wide shortage of amoxicillin over the last couple of years and it would have been useful to bring in some of that real-time data too.

We thank the reviewer for this positive assessment of our work. In order to further ground our study on observed data, we have added a few sentences describing the recent Europe-wide amoxicillin shortage to the introduction, and referencing a very recently published paper, as follows:

L. 77-82 "A shortage of amoxicillin and its combination with clavulanate was announced by the European Medical Association in January 2023. A recent study analysing 49 databases across 18 European countries found a subsequent drop in amoxicillin use in 55% of these databases, and a drop in amoxicillin-clavulanate combination use in 69% of cases, with the decline most evident in primary care (Pineda-Moncusi et al, 2025)".

The disclaimer for conducting this review is that I do not have mathematical modeling expertise. Hence I reviewed this from a pragmatic clinical and practice perspective. My comments below are in order of appearance in manuscript and (for expediency) not in order of importance.

Abstract

The link to bacterial consequences are hypothesized but not backed by evidence yet.

Indeed, the bacterial consequences of antibiotic shortage are not backed by evidence yet. This is why we mentioned "potential major consequences". But knowing that antibiotic consumption is one of the main drivers of resistance patterns in *S. pneumoniae*, we have good reason to believe that antibiotic shortages do impact bacterial ecology. We modified the abstract accordingly in the revised version of the manuscript :

L. 27-28 "Their impact on individual health and bacterial ecology is unknow, but it could be significant, and there is a lack of epidemiological evidence."

Introduction

Generally well-written and brings in the existing body of literature on antibiotic shortages. Lines 75-79 The existing literature, mainly from high income settings and hospitals describes a limited number of studies that have data on impact of shortages on infection and points to a mixed data. What can be said is that the increasing occurrence of shortages of Access and Reserve category agents is compromising optimized antibiotic care and causing knock on shortages of substitute agents. It also depletes existing antibiotic stewardship programmes where they exist.

We thank the reviewer for drawing attention to these important aspects of antibiotic shortages and their implications for optimized antibiotic care. In response, we have incorporated the concepts of Access and Watch categories into the main text as follows:

L. 71-73 “In some contexts, this could promote the use of macrolides, which belong to the WHO AWaRe framework 'Watch' category due to their higher resistance potential.”

We have expanded our discussion to address how shortages can undermine established stewardship efforts, as follows :

L. 669-673 “Moreover, antibiotic shortages can significantly undermine stewardship programmes by forcing prescribers to deviate from evidence-based guidelines toward less optimal, broader-spectrum alternatives. In settings where such programmes are well-established, these disruptions risk undoing years of progress in optimising prescribing practices.”

Lines 80 to 87 The impact of shortages on any given infection or resistance epidemiology is not well understood, it is not specific to pneumococcal disease. Did the dynamic modelling work on a hypothetical shortage scenario or was it developed using the existing global shortage of amoxicillin?

We appreciate the reviewer’s thoughtful question. Our modelling was based on hypothetical shortage scenarios, rather than directly replicating the recent global shortage of amoxicillin. However, the range of beta-lactam shortages we explored was informed by observations reported in the literature, to ensure plausibility and relevance. In addition, the scenarios were designed in close collaboration with clinicians to capture as accurately as possible how prescribing practices adapt in real-world settings during a shortage. In response to this comment, we revised the manuscript as follows:

L. 728-730 “We defined four antibiotic allocation strategies under shortage, based on observed behaviours and national recommendations reported between 2022 and 2024[6,9,11,12], in collaboration with clinicians to reflect real-world prescribing adaptations.”

Results/methods

In a real case scenario reducing dose would probably be more relevant if there were substandard formulations.

We agree with the reviewer. This scenario is the most theoretical one, as we are aware that all the investigated values for doses reduction percentage are not feasible in real life due to formulation. However, considering a 50% shortage, this could correspond to a change from 3 doses of 1000mg/day to 3 doses of 500mg/day , a relevant scenario from a standard formulation point of view. We have revised the Discussion section of the manuscript as follows:

L. 638-642 “Reducing the daily dose (S3) are the most theoretical strategies among the investigated ones, they would rely on the availability of substandard dose formulations for purchase, and depending on the investigated values some of them seam unrealistic. However, the 50% reduction (3 × 500 mg/day instead of 3 × 1000 mg/day) is relevant and feasible from a formulation perspective.”

For shortages the most common option for pediatric medicine is switching to a different agent, using adult formulations and crushing/administering.

Yes, in clinical practice, from a prescriber's perspective, the preferable option was to adapt adult formulation to paediatric doses. However, national recommendations from several countries, such as France, advise switching to another molecule when such adaptation was not possible. This alternative approach (strategy S4 in our paper) has been documented in the literature, as referenced in our introduction (Line. 77), where studies have reported an increase in the use of alternative treatments during shortage periods. We included some additional discussion on that aspect as follows :

L. 642-645 "Switching to macrolides (S4), although undesirable for resistance, would likely occur in practice during shortages to ensure effective treatment of infected children, even though adapting adult formulations to paediatric doses is generally the preferred (but not always available) option."

Line 566 – error message for figure needs to be corrected

We thank you for highlighting this and have corrected the error message.

Discussion

Line 421 The impact of shortages also depend on stewardship capabilities, healthcare infrastructure, procurement and responsiveness of the health system to dealing with the shortage. So to develop a model that purely considers the dosage and switching is a limitation. The model works for high income countries with universal health coverage and assumed otherwise good access to healthcare and antibiotics. The model would be very different for low-resource settings where access to medicines, including antibiotics, can be via informal routes, with higher rates of exposure to substandard and falsified medicines, and paediatric populations with different vulnerability and epidemiological (and vaccination) profile for pneumococcal diseases.

We thank the reviewer for raising this important point. We agree that our model is applied to high-income settings with well-established healthcare infrastructure and generally good access to antibiotics and that the situation would be probably strongly different in other settings. To address this limitation, we have added a dedicated paragraph in the Discussion section, as follows:

L. 606-613 "In this study, we modelled beta-lactam shortages in the context of a well-functioning healthcare systems with good access to antibiotics, as typically observed in high-income countries with universal health coverage. This approach does not account for differences in stewardship capabilities, healthcare infrastructure, procurement processes, or the responsiveness of the health systems, which may substantially influence the impact of shortages. In low-resource settings, where access to alternative treatments is more limited, informal distribution channels are common, and exposure to substandard or falsified medicines is higher, the dynamics of shortages would differ markedly."

The capability of stockpiling, contractual pricing also influences the response to dealing with shortages. So on lines 555 it would be very helpful to identify what the authors mean by 'establishing clear public health priorities' bearing in mind this needs to be contextualized.

We thank the reviewer for this pertinent suggestion. We agree that factors such as stockpiling capacity and contractual pricing strongly influence the ability to respond to antibiotic shortages. Regarding line 555, by "establishing clear public health priorities" we mean that health

authorities should define, in advance, context-specific priorities that balance individual and population benefits. Our findings show that no single strategy systematically reduces both resistance and invasive pneumococcal disease, and choices may differ according to epidemiological and economic circumstances at the country level. Thus, “clear public health priorities” refers to tailored, practical recommendations adapted to each context and available resources, in order to avoid confusion and inconsistency during shortages. This is in line with the WHO, which recommended tailoring antibiotic treatment based on local resistance patterns. While defining national guidelines is outside the scope of our work, our results provide a framework to help policymakers anticipate the likely consequences of alternative strategies. We have expanded the last paragraph in the Discussion section to clarify what we mean by “establishing public health priorities”, as follows :

L. 647-659 “In practice, the response to shortages depends not only on epidemiological considerations, but also on structural and economic factors, and the ability of healthcare systems to enforce stewardship measures. Our model shows that no single strategy consistently achieves both the reduction of invasive disease and the limitation of resistance, and that recommended approaches may vary according to local resistance levels. In this context, national health authorities should anticipate and define, in advance, context specific objectives that balance individual and population benefits and serve as a basis for practical recommendations. Such prioritization requires considering several country-specific factors, including the local prevalence of resistant strains, the availability and affordability of alternative antibiotics, the capacity for stockpiling, and healthcare organization.”

Stewardship only features in the discussion and then in concluding paragraph. Could the authors have considered building in the consequence of effective stewardship into the model?

We thank the reviewer for this interesting remark. While stewardship is mainly discussed in the discussion section, we would like to clarify that it is addressed throughout the paper via the different strategies tested (S1–S4), as explained in the Discussion with this sentence: “Strategies can be seen both as a consequence of hypothetical recommendations implemented in response to shortages and as a concrete impact of shortages in the population.” So, these strategies can be interpreted as stewardship interventions that might be implemented in response to beta-lactam shortages.

Existing evidence shows that managing antibiotic shortages whilst benefiting from stewardship can also compromise it leading to knock on effect on quality of antibiotic use in health systems. So the ecological and epidemiological consequences can be beyond a single pathogen or diseases/population.

Yes, thank you for this comment. We focused in this study on one bacteria *S. pneumoniae* and in associated invasive diseases outcomes. Of course, shortages, by changing prescription behaviours, also impact other disease that are not treated in time due to shortages. By changing rates od prescriptions of broad-spectrum agent such as macrolides, they can also affect the ecology of other bacteria. To reflect this important and wider ecological and epidemiological perspective, we added the following sentence in the Discussion section:

L. 622-626 “Also, we focused specifically on *S. pneumoniae*, a prevalent community pathogen, and limited our analysis to invasive disease outcomes. Beyond pneumococcal epidemiology, the modelled shortages would affect colonisation, other infection dynamics and resistance of other circulating community bacteria such as *H. influenzae* and *E. coli*. The model could be

extended to other pathogens and diseases to assess their evolution under antibiotic shortages."

Minor suggestions and typos
Can remove 'both' from line 27
Line 62 change amoxicillin forms to formulations
Line 75 should be changed to USA
Throughout replace multivariate with multivariable

We thank the reviewer for identifying these points. We have revised the manuscript accordingly.

Reviewer #3

Ritika Kondal:
I co-reviewed this manuscript with one of the reviewers who provided the listed reports. This is part of the Nature Communications initiative to facilitate training in peer review and to provide appropriate recognition for Early Career Researchers who co-review manuscripts.

General Comments: Well written. Interesting and meticulously conducted study. Model development was done with consideration of actual scenarios like, resistance, fitness cost and competition parameters. This gives an edge to the complete mathematical modelling study which is not backed by actual data.

We appreciate the reviewer's positive feedback on our work.

Introduction:
Line 79: One line about how shortages can impact carriage prevalence can be added. It will give context to the model researchers have developed, as they have taken these parameters in model development.

Thanks for this pertinent remark. Shortages may indeed directly affect pneumococcal carriage prevalence, as decreased antibiotic availability (e.g. during beta-lactam shortages) leads to fewer colonized individuals clearing their carriage through treatment, allowing strains to persist and circulate more widely in the community, and resulting in increased carriage prevalence. We agree that explaining this link with carriage prevalence is important. As this mechanism is implemented in the model's assumptions, we have added a clarifying sentence in the Method section rather than the Introduction, to keep the latter focused on general evidence. The following sentence was added:

L. 695-698 "In this framework, antibiotic use shapes both overall pneumococcal carriage prevalence (through its effect on clearance of colonization) and the distribution of resistance among circulating strains (through selective pressure)."

Line 85: Can be written different prescribing strategies instead of "patterns" as through the paper, authors have used the term strategies

We thank the reviewer for this helpful remark. We agree that "strategy" is more appropriate than "pattern" and have revised the text accordingly for consistency throughout the manuscript.

Methods:
Line 569: After looking at the colonization status, described later and in the results also, this line is contradictory. If exposed children will always have resistant strains exposure, then why to mention susceptible strains?

In our model, antibiotic exposure is independent of colonisation status. Therefore, children receiving antibiotics may be non-carriers (susceptible) or carriers of strains with different levels of resistance (from susceptible to resistant). This is why we also mention susceptible strains in this context. We hope this clarifies your concern and addresses your question.

Line 620: Steady state of which index/ parameter was assumed?

Thank you for this remark. As also pointed out by another reviewer, the description of our calibration method was not sufficiently clear. In response to your specific comment, we now clarify that the transmission parameter and fitness costs were calibrated to achieve a steady pre-shortage baseline in both prevalence and resistance distribution. We have accordingly revised the “Calibration of transmission parameters” (L. 756-810) subsection in the Methods to provide a more detailed description. Specifically, the modified sections include: (i) the calibration of antibiotic-induced decolonization rates (L. 762-792), and (ii) the calibration of transmission parameters and fitness costs (L. 793-810).

Line 624: full form of PNSP needs to be given before

Thank you for pointing this out. The definition of PNSP was initially introduced in the Results section and at the end of the Methods section. To improve clarity, we have now moved the “Shortage simulation” subsection to the start of the Methods section so that all variables, including PNSP, are defined earlier in the text, as suggested.

Meaning of gamma and lambda is not explained in the methods section. It’s explained in figures legends. But needs to mentioned in the method section for better clarity.

We agree that all parameters need to be defined in the text (and not only in the Figure legends), and have now added a description of λ and γ in the “Model description” subsection of the Methods:

L.693-694 “Antibiotic exposure ends at a rate γ, with $1/\gamma$ corresponding to the mean treatment duration.”

L. 701-702 “The colonization status is characterised by the strain’s resistance levels to both penicillin and macrolides ,as well as by its duration, represented as 1/λ, where λ denotes the clearance rate”

Line 664: Are authors want to say about rate of infection in child? The sentence seems incomplete.

Thank you for this comment. We realize that the term “rate per child per year” was ambiguous. In fact, we refer to the rate of beta-lactam consumption frequency per child per year. We have revised the sentence to make this clearer, as follows :

L. 734-736 “Strategy 1 (S1), reducing beta-lactam consumption frequency, which reduces the consumption frequency rate from 0.95 to 0.71, 0.48, 0.24 and 0.05 per child per year.”

Line 675- 678: The sentence should come before in the method section for better clarity.

To improve clarity, we have now moved the shortage simulation subsection to the start of the Methods section so that all variables, including PNSP, are defined earlier in the text, as you suggested.

Results:

Fig 2: Colour of the figure needs to be corrected. (grey scale is not visible in the figure)

Thank you for this comment. However, Figure 2 does not use a grey scale. Could you please clarify if you were referring to another aspect of the figure’s readability? We would of course be happy to adapt it to ensure full clarity.

Line 161-164: How could strategy 1-3 will have an effect on macrolide exposure? Not able to understand.

Thank you for this comment. Strategies S1–S3 indeed only affect beta-lactam exposure and have no impact on macrolide use. We realize that our transition sentence “These first three strategies have no impact on macrolide exposure” may have been confusing, and we have removed it to avoid any ambiguity.

Discussion:

Line 519-527: Its repetition of the results from Fig 6. Possible explanation for the same could be given.

Thank you for this remark. Our intention with this section was not simply to repeat the information displayed in Figure 6, but to provide an additional perspective by translating the model outputs into absolute case numbers for the entire French population, rather than only reporting incidence per 100,000 inhabitants. However, we acknowledge that this added explanation was largely redundant and did not substantially improve the clarity of the manuscript. We have therefore removed the section in the revised version.

This study outcomes can predict the impact of different strategies in HIC countries where a defined system and protocol is followed for management of infection. However, when it comes LMIC’s due to high burden of infection, most of the primary and secondary care facilities do not have the resistance mapping labs. So, variability of proportion of resistance strains cannot be applied to LMIC settings. Authors should mention the applicability of the study in HIC.

We thank the reviewer for these pertinent remarks. In the revised version of the manuscript, we have addressed them through a dedicated paragraph in the Discussion section:

L. 606-613 “In this study, we modelled beta-lactam shortages in the context of a well-functioning healthcare systems with good access to antibiotics, as typically observed in high-income countries with universal health coverage. This approach does not account for differences in stewardship capabilities, healthcare infrastructure, procurement processes, or the responsiveness of the health systems, which may substantially influence the impact of shortages. In low-resource settings, where access to alternative treatments is more limited, informal distribution channels are common, and exposure to substandard or falsified medicines is higher, the dynamics of shortages would differ markedly.”

Reviewer #2:

The manuscript fills a gap in understanding the impact of policies with this natural occurring experiment. The authors elicit a relevant set of policy options and outcomes and those from the data accrued. This study also sheds some understanding on the nuances and implications when we think of reducing antibiotic prescribing, and its impact on antimicrobial resistance and

infection burden. The authors considered alternative endpoints to illustrate the impact of interventions on different realms of policy.

We thank the reviewer for this positive assessment of our work.

The quality of the data is good and suited to be used in a simulation for European countries. This is also only possible given the quality of ECDC data. The sensitivity analysis shows that main drivers of outcomes are considered in the analysis and interventions seem to have a linear and expected effect on outcomes.

Some of the findings speak to the broader discussion of appropriate use and prescription of antibiotics in other regions.

With regard to the wider implications of our work for antibiotic stewardship and rational use, we would like to highlight that this aspect is addressed in our discussion (L. 614-618). There, we position our work within the WHO global efforts to prevent antibiotic resistance while promoting rational antibiotic use. Specifically, we state: "This study was achieved in the context of increasing drug shortages but is also in line with the World Health Organization global efforts to prevent antibiotic-resistant and multidrug-resistant infections while promoting the rational use of antibiotics. Indeed, by focusing on beta-lactam shortages as part of rational antibiotic use, our proposed strategies (S1-S4) provide valuable insights for international antibiotic stewardship initiatives."

However, there is very limited data from those and also extreme scenarios that make non-linearities more likely to emerge. These caveats could perhaps be considered in a couple of phrases in lines 537 onwards.

In a context of lack of data, we aimed here at developing a large-scale simulation to evaluate the ecological impact of the different policies if they were systematically implemented at a country level. In our simulations, we explored a large range of scenarios including some that may be unrealistic or impossible to set up in practice (e.g. non-available formulations). Some of these scenarios may indeed lead to non-linearities. In this context, we acknowledge that the evaluated scenarios can sometimes be speculative and that’s the reason why the model was then applied to real case studies at the European level.

I think the authors do a fair assessment of the extent to which their conclusions would be valid and can inform decision making, also leaving room for the normative assessment of policy aim ranking. I also believe the author’s findings speak to the broader discussion of appropriate use of antibiotics. This highlights that stewardship capacity could make use of associated interventions that mitigates these undesirable effects and optimises prescribing.

We sincerely thank the reviewer for this very positive and encouraging assessment. We are pleased that our analysis is seen as contributing both to the evaluation of policy relevance and to the broader discussion on the appropriate use of antibiotics.

Suggested improvements

1.It could be useful for some readers to divide figure 6 into high-vs-low prescriber groups. It could make it easier to glance trends that are spelled in the text. It would also make some comparisons interesting, as I imagine Denmark being in the low-prescribing group yet exhibits some trends similar to high-prescribers.

Thank you for this helpful suggestion. We have reorganized Figure 6 according to countries overall antibiotic consumption, sorting them from highest- to lowest-prescribers, rather than in alphabetical order. We believe this indeed improves the readability of trends and facilitates the comparisons highlighted in the text.

2.Please specify the "initial prevalence" on line 509.

Thank you for this remark. The initial prevalence refers to the baseline pneumococcal carriage prevalence, which was set to 0.52 in our model. Following manuscript revisions, this sentence no longer appears in the main text in its original form. However, this parameter is now clearly specified in the revised Supplementary Table 2, which has been restructured for improved clarity and readability.

3.Lines 498-511 mention the limitations of using the French data and how the sensitivity analysis showed that the greatest impact is accounted for in the simulations. However, it does not mention the fact that France was regarded as a high-prescriber yet used to draw conclusions on low-prescriber countries.

Thank you for this insightful comment. You are correct that the French data used in our model reflect the context of a high-prescribing country. However, our European-level analyses did account for between-country variations in the initial overall resistance and multi-resistance levels, as well as in beta-lactam and macrolide exposure, thereby limiting the potential bias associated with France's high-prescriber status. Additionally, our sensitivity analysis confirmed that the parameters with the greatest impact on the recommended strategy were initial resistance and multi-resistance levels.

We would like to clarify that most model parameters were not sourced exclusively from France. Biological parameters such as colonization duration, carriage prevalence, and invasiveness rates were derived from European literature (Sweden, England). These parameters are largely age-dependent and comparable across European high-income countries with similar healthcare systems, although they may differ in low- and middle-income settings. The assumed treatment duration of seven days is consistent with standard European clinical recommendations.

The only parameter sourced specifically from French data is the MIC distribution among penicillin-non-susceptible strains. We acknowledge uncertainty about whether this distribution fully represents other European countries, particularly lower-prescribing settings. To address this concern, we conducted two sensitivity analyses. First, we expanded the multivariate PRCC analysis (Supplementary Figure 3) exploring variations around the French MIC distribution values. This analysis examined how local variations in the distribution affect model outcomes (resistance prevalence and invasive disease incidence) under different shortage strategies and showed that such variations had minimal impact on strategy rankings. Second, to test more extreme distributions, we used completely different MIC distributions sourced from ATLAS surveillance data. While these data have important limitations (lack of age stratification, heterogeneous sample sources, and limited sample size leading to combine data spanning 2004 to 2023), they provide plausible alternative distributions to test our model's robustness. We generated Supplementary Figure 5 showing recommended strategies using these alternative distributions. Although these extreme distributions slightly shifted the boundaries between recommended strategies, the changes were minimal and the overall strategy rankings remained consistent.

To conclude, the fact that France is a high prescriber should not substantially affect their values and thus should not change our main conclusions. In addition to the new sensitivity analyses, we have restructured Supplementary Table 2 to clarify parameter values and sources for both the French and European analyses. We have also restructured the paragraph in the Discussion to explain more clearly the source of the data and to highlight the associated limitations:

L. 558-570 “Country-specific differences were modelled by varying antibiotic consumption frequency and initial resistance levels. Other parameters, including treatment duration, pneumococcal carriage prevalence, and duration of colonization, were held constant across countries based on European studies. These parameters are largely age-dependent and comparable across European high-income countries with similar healthcare systems; the treatment duration of seven days is consistent with standard European clinical recommendations. The only parameter sourced specifically from French data was the MIC distribution among penicillin-non-susceptible strains. We acknowledge uncertainty about whether this distribution, derived from a high-prescribing country, fully represents lower-prescribing European settings. However, sensitivity analyses exploring different initial MIC distributions (Supplementary Figure 5) demonstrate that these distributions are not a critical driver of strategy rankings. The multivariate PRCC analysis (Supplementary Figure 3) confirmed that initial overall resistance and multi-resistance prevalence are the most influential parameters.”

4.Lines 537-545 rightly highlight the implications of this study for stewardship interventions. This highlights the importance of associated intervention that mitigates the initial effects from stewardship which could be unpopular.

Thank you for this positive remark. We agree that stewardship interventions may initially be perceived as unpopular or challenging, and that accompanying measures are important to mitigate these early effects and support their implementation.

5.Typo on line 158 : “and per day”

6.Typo on line 202: “ all S. pneumoniae”

Thank you for pointing these typos, which have been removed.

7.Line 566 on materials and methods, model description, the reference to figure 1 shows an error.
8.Line 625 on materials and methods, model description, reference to supplementary methods shows an error.
Thank you for pointing out these errors. The incorrect reference to Figure 1 and the incorrect reference to the Supplementary Methods have been corrected.

Reviewer #4 :

This study by Maurin et al develops a novel varying resistance level model of beta-lactam and macrolide resistance in S. pneumoniae. They explore a highly pertinent question around the impact of antibiotic shortages and develop multiple interesting results that will be useful to policy makers and in considering the future impacts of these increasingly frequent events. I have some points of clarification and a few recommendations in terms of putting the results in context but otherwise believe that this a solid piece of work. My only major concern is around understanding exactly what was done in terms of model fitting but I hope this is only a clarification issue.

We thank the reviewer for this positive assessment of our work and for the insightful comments on clarifying the parameter-estimation procedure. We appreciate the time dedicated to reviewing our manuscript and the constructive suggestions, which have helped us improve the clarity of the paper. In response, we have revised the Methods section to provide a clearer explanation of the calibration procedure, with the aim of making the fitting process as transparent and reproducible as possible.

Major

- Should more be made of the impact on MDR-strain increases? What are the treatment options for these infections? Surely this dual resistance is the most worrying compared to the mono-resistances and is the one to focus on compared to IPD increase?

We appreciate this important comment regarding multidrug-resistant (MDR) *S. pneumoniae*. In our study, MDR refers to dual resistance to both beta-lactams and macrolides, which is indeed a more clinically challenging and concerning scenario than mono-resistance. For such infections, therapeutic alternatives include respiratory fluoroquinolones, combination therapy, and in some cases the use of newer agents such as ceftaroline or tedizolid (Van Bambeke et al., 2007). While these options can be effective, fluoroquinolones raise concerns about fostering antimicrobial resistance and have suboptimal safety profiles for widespread community use, whereas newer molecules remain limited in availability and are generally reserved for highly resistant cases.

We fully agree that dual resistance deserves greater emphasis as a key threat. We therefore added a sentence in the introduction highlighting MDR as the greatest therapeutic challenge and expanded the discussion with a dedicated paragraph explaining the clinical and public health implications of MDR *S. pneumoniae* in more detail, as follows :

L. 86-92 "An important concern is also multidrug resistance, which can lead to treatment failures or delays. Alternative therapeutic options for multidrug-resistant infections include respiratory fluoroquinolones, combination therapy, and newer agents such as ceftaroline. However, these alternatives raise significant concerns: fluoroquinolones have suboptimal safety profiles for widespread community use, while their broad-spectrum activity can drive antimicrobial resistance through co-selection of resistance determinants, thereby amplifying resistance at the population level."

L. 592-600 "It is important to distinguish between the implications of penicillin-resistance, macrolide-resistance, and dual resistance. Amoxicillin is the first line and most affordable option for pneumococcal infections; resistance therefore directly affects standard care. Macrolide resistance, though less critical in some situations, is still recognized as a major global issue; *S. pneumoniae* resistant to macrolides was recently included by WHO (2024) among its priority pathogen–antibiotic combinations. Dual resistance to both beta-lactams and macrolides remains the most worrisome scenario, as it leaves only fluoroquinolones or newer, less available agents as options. This situation increases costs, safety risks, and the likelihood of further resistance selection, making MDR the greatest therapeutic and public health challenge."

- Throughout care needs to be taken that fitness cost and relative fitness are not used incorrectly. I think that a "fitness cost of 0.995" for example should be a "relative fitness of 0.995". Also Table 1 should be relative fitness not cost?

We thank the reviewer for raising that point. In our model, the parameter f_k is indeed mathematically a relative fitness. We have changed spelling accordingly in the manuscript, changing the name fitness cost by relative fitness when appropriate.

- The calibration section is unclear – paragraph starting line 608. What is the likelihood / L-BFGS algorithm / initial conditions? why only after 1 year do you do the fitting? normal distribution of what? I don't understand how this matches then what is done in the next paragraph? How can you fit the transmission / fitness cost without antibiotic exposure? Line 622 – what change? Why is least squares now being used no the L-BFGS?

Thank you for raising this point about the calibration procedure. Following your suggestions, we have completely rewritten the "Calibration of transmission parameters" (L. 756-810) subsection in the Methods section to provide a more detailed and transparent explanation of our methodology. Below, we address your points one by one:

We assumed observed carriage prevalence and serotype distributions to follow a normal distribution around our model predictions $\widehat{p_t}(\theta)$ and $\widehat{d_t}$. This Gaussian error assumption explains why we alternatively referred to "maximizing the likelihood" and to "minimizing least squares". Indeed, under normal error assumptions, minimizing the negative log-likelihood is mathematically equivalent to minimizing the sum of squared differences between observations and predictions. To avoid any confusion, in the main text we now refer exclusively to "minimizing least squares".

Regarding the optimization algorithm, we use the Limited-memory BFGS (L-BFGS-B) method, which efficiently handles bound-constrained optimization problems by approximating the Broyden-Fletcher-Golfarb-Shanno algorithm while respecting parameter constraints.

We've corrected the misleading statement about fitting "after 1 year." In reality, we assumed that carriage prevalence and resistance distributions have remained approximately stable since 2021, and we calibrated transmission parameters and fitness costs by minimizing the distance between simulated dynamics over a five-year period and the 2021 observed values, rather than starting the fitting process after one year.

Finally, we clarified the previous unclear description of antibiotic exposure during calibration in the 'calibration of antibiotic-induced decolonisation rates' subsection. In practice, our calibration of transmission rates β and fitness costs f_k accounts for community-level antibiotic exposure. Specifically, we calibrated these parameters to ensure that carriage prevalence and resistance frequency remained stable over time under background antibiotic pressure. Only after establishing these baseline parameters did we simulate the specific intervention scenarios with their respective dosing regimens and durations.

- More needs to be included in the introduction and discussion on the beta-lactam / macrolide / IPD treatment balance. The introduction needs more on macrolide use and resistance. Isn't MDR the biggest issue as then there are no (?) treatment options? What is the difference between beta-lactams and macrolides in terms of cost / efficacy / mortality / AWaRE classifications?

Thank you for this valuable comment. We agree that the balance between beta-lactam and macrolide use, and its impact on IPD and resistance, is a crucial point. However, we chose not to present this aspect in the introduction, as it reflects one of the main conclusions of the paper. For this reason, we preferred to address it in the discussion section, where it is already

explicitly stated: "This highlights the trade-off between individual benefits (usage of antibiotic to limit incidence) vs. population benefits (antibiotic use increases the resistance in the population). A complex balance therefore needs to be struck between reducing the risk of resistant infections and managing the overall disease burden."

Regarding macrolides, we have expanded the introduction as suggested: beta-lactams are relatively inexpensive and mostly belong to the WHO AWaRe "Access" group, whereas macrolides are more costly and classified as "Watch" antibiotics due to their higher resistance potential. :

L. 64-67 "Amoxicillin is a first-line therapy for most paediatric bacterial invasive infections[10] . It is an effective and relatively inexpensive beta-lactam drug available in generic formulations, and classified by the WHO AWaRe framework in the "Access" category, reflecting its importance as a safe first-line treatment with relatively low resistance potential."

L. 71-73 "In some contexts, this could promote the use of macrolides, which belong to the WHO AWaRe framework 'Watch' category due to their higher resistance potential."

Finally, we agree that MDR constitutes the greatest concern, as it leaves only fluoroquinolones, combination regimens, or newer agents such as ceftaroline or tedizolid as alternatives. These remain less accessible and raise further resistance and safety concerns. As noted in our response to your earlier major comment, we have addressed this issue by adding clarifications in the Introduction (L. 86-92) and by revising the Discussion accordingly (L. 592-600).

- The writing is sloppy in places – a grammar check needs to be done throughout – e.g. line 114 "a" is missing before rate, line 599 "despite colonized" needs correcting. The discussion especially needs a careful read through. Similarly there are some "Error missing source" etc.

Thank you for pointing out these grammatical errors. We have carefully proofread the text to try and remove as many as possible. The "Error missing source" are all corrected, thanks.

Minor

Abstract: I think more needs to be included in here about the main analysis which is based on the French situation with less focus on the country comparisons (only one figure on this and it uses many French parameters).

We agree that the results section of the abstract may have been somewhat unbalanced. Indeed, most of our analyses (Figures 2-4) are based on the French situation, which we used primarily to illustrate and introduce the mechanisms underlying the different shortage strategies. Figure 5, however, does provide an extension of our results under different parameter settings, and is related to the between-country comparison in Figure 6. We have revised the Abstract by adding information on the key parameters to consider when identifying the optimal strategy, and by shortening the sentence that described country-specific results as follows :

L. 37-44 "We found heterogeneous impacts of allocation strategies, amplified at high shortage intensity. The adverse outcomes of shortages increased with baseline antibiotic consumption and the main determinants of the optimal strategy were the initial prevalence of resistance. Reducing beta-lactam frequency most effectively limited resistance, lowering penicillin-non-susceptible and multidrug-resistant strains by up to 21% in Spain during a 50% shortage. The optimal strategy for minimizing IPD incidence was country-dependent: either lowering the daily dose or beta-lactam-to-macrolide substitution. However, the latter significantly increased macrolide resistance, reaching up to 26.2% in Denmark, for a 50% shortage."

Abstract:

I think "consequences" needs to be clearer – add into previous paragraph?

Thank you for this comment. We agree that the term "consequences" was too vague. As detailed earlier in the abstract, our analyses focus on the impact of beta-lactam shortage management on pneumococcal resistance and the incidence of invasive pneumococcal disease (IPD). To make this explicit and to underline that we are referring to negative outcomes, we have revised the sentence as follows:

L. 38 "The adverse outcomes of shortages increased with baseline antibiotic consumption."

Line 78: Ref 15 says in abstract "Multivariable analyses identified significant associations of ABR with country-level selecting antibiotic sales, but only in fluoroquinolone-resistant-Escherichia coli, fluoroquinolone-resistant-Pseudomonas aeruginosa, and carbapenem-resistant-A baumannii." so not for S. pneumoniae?

Thank you for this comment. In Ref. 15, two types of associations were analysed. The abstract highlights only the drug–bacterium pair analyses, where significant associations were found for fluoroquinolone-resistant E. coli, fluoroquinolone-resistant P. aeruginosa, and carbapenem-resistant A. baumannii. However, in their broader analysis based on global antibiotic sales (rather than specific drug–bug pairs), the authors report that ABR rates in *S. pneumoniae* pairs were significantly associated with overall antibiotic consumption. This is the result we were referring to.

Introduction: overall couldn't shortages be a good think in terms of reducing overall resistance?

From a population-level perspective, an unplanned shortage of antibiotics reduces overall usage, which can indeed reduce the selective pressure that drives resistance. However, at an individual level, shortages risk leaving infections untreated or inadequately treated, which can worsen patient outcomes. Additionally, when first-line drugs are unavailable, clinicians often switch to second- or third-choice antibiotics, which are typically broader-spectrum agents, and this can increase resistance in those drug classes. Although reducing unnecessary antibiotic use is beneficial for controlling resistance, sudden shortages are an overly drastic measure that may endanger patients and simply shift resistance pressure onto broad-spectrum drugs. To address this comment, we revised the manuscript to include the following discussion:

L. 660-668 "Although our model suggests that antibiotic shortages may reduce the selective pressure driving resistance, this apparent benefit is outweighed by an increased burden of severe infections and contributes to preventable morbidity and mortality in children. Shortages also carry an additional economic cost, as second-line alternatives such as macrolides are generally more expensive, and poorly treated infections often lead to increased healthcare utilization, including hospital admissions. Overall, shortages cannot be considered beneficial: the unintended reduction in resistance is outweighed by the immediate clinical and economic costs. Instead, the aim of reducing antibiotic use should be achieved through controlled stewardship strategies rather than through unpredictable shortages."

Line 76: I don't understand the link to resistance here.

We agree that this was not explicit enough. The link to resistance at this point refers to the use of alternative antibiotic molecules when the recommended ones are unavailable; for instance, drugs falling into the Watch or Reserve categories of the WHO AWaRe classification. Such substitutions are associated with a higher risk of fostering resistance, either by driving multidrug resistance in *S. pneumoniae* itself or by promoting resistance in other clinically important bacteria–antibiotic pairs. In other words, deviating from first-line recommendations during shortages can accelerate the selection and spread of resistant strains, which is the mechanism we intended to highlight in this sentence. In response to this comment, we revised the manuscript as follows:

L. 82-84 "As a consequence, antibiotic shortages may increase the risk of diseases[14] but also constitute a threat in terms of spread of antimicrobial resistance[15], especially with regard to the substitute antibiotics used during shortages."

Introduction: Please include more on how policymakers / prescribers could or might be influenced by modelling results showing the balance between resistance and disease. Who makes decisions following shortages on how it affects use? Either here or in the discussion.

We thank the reviewer for this suggestion. In order to address this point and also to incorporate Reviewer 1's request for greater clarity on what we meant by 'establishing clear public health priorities', we have added a dedicated paragraph in the Discussion section, as follows:

L. 647-659 "In practice, the response to shortages depends not only on epidemiological considerations, but also on structural and economic factors, and the ability of healthcare systems to enforce stewardship measures. Our model first highlights that shortage have indeed consequences at the population level. Second, it shows that no single strategy consistently achieves both the reduction of invasive disease and the limitation of resistance, and that recommended approaches may vary according to local resistance levels. In this context, national health authorities should anticipate and define, in advance, context specific objectives that balance individual and population benefits and serve as a basis for practical recommendations. Such prioritization requires considering several country-specific factors, including the local prevalence of resistant strains, the availability and affordability of alternative antibiotics, the capacity for stockpiling, and the organization of healthcare systems to support stewardship measures. By highlighting the trade-offs between infection burden and resistance, our modelling results can support policymakers in drafting clear guidance during shortages and help prescribers understand the implications of adapting their practice in line with national priorities."

Introduction: I think this also could do with a sentence or two about the novelty of the MIC variation included in the model – setting up that S. pneumoniae has variation in this and the importance for resistance transmission / antibiotic exposure impact. Moreover why this variation is more for beta-lactams than macrolides.

Thank you for this suggestion. We had previously explained the rationale for modelling resistance differently across drug classes in the Methods: "pneumococcal resistance to beta-lactams is described according to the minimum inhibitory concentration (MIC), given that successive mutations are responsible for resistance to beta-lactams[34]. The model includes eight levels of *S. pneumoniae* penicillin susceptibility (0.063, 0.125, 0.25, 0.5,1,2,4,8 mg/L) representing increasing resistance levels. […] In Europe, macrolide resistance mechanism is predominantly linked to an enzymatic modification of the target (MLS_B resistance)[30], allowing only two resistance states: Susceptible or Resistant." In line with your comment, we have now

added a sentence in the Introduction to better highlight the novelty of this modelling approach :

L. 97-101 “Our model captures resistance dynamics by incorporating eight distinct MIC levels for penicillin resistance[17] and two resistance states for macrolides[18]. This fine-scale structure enables a more precise evaluation of antibiotic exposure, dosage effects, and fitness costs across the two drug classes.”

Results

Figure 1: what are lambda / phi / delta? Either include legend or refer to table which describes them

Thank you for this remark, we fully agree that all parameters should be defined in the text. The explanation of the Φ parameter was already provided in the figure legend; we have now added the missing explanations for λ and δ, wich corresponds to the natural decolonisation rate and the antibiotic-induced decolonization rate, respectively :

“The antibiotic-induced decolonization rate to beta-lactams $\boldsymbol{\delta_k^{Amox}}$ or macrolides $\boldsymbol{\delta_k^{Macro}}$ depends on the daily administered dose and the bacterial resistance level of the carried strain to the specific administered antibiotic.Antibiotic exposure ends at a rate $\gamma^{Amox}$and γ^{Macro}, respectively. β represents the pneumococcal transmission rate, multiplied by a fitness cost f depending on the level of strain resistance. λ corresponds to the natural decolonisation rate.”

Figure 1: k is numeric I think so it should be k is a member of {1,2,..,8} not CM1 etc

Yes, you are right. We have corrected this as follows : "$C_{CMIk\_m}, k \in \{1, 2, \dots, 8\}$ and $m \in \{S, R\}$." Thank you for bringing it to our attention.

Line 110: evidence for a fitness cost? And its potential size?

Thank you for pointing this out. We agree that references documenting the evidence of fitness costs for resistant strains were missing. We have now added two relevant references (L. 128): Domenech de Cellès et al. (2015) and Krishna et al. (2025).

Regarding the potential size of the fitness cost, this is addressed in the Discussion: “The estimated fitness values ranged from 0.92 to 0.99. To our knowledge, this is the first model to incorporate a landscape of fitness costs on different levels of resistance. The resulting weighted average of the estimated fitness costs when considering the MIC distribution observed in France was 0.944. it aligns with previous estimates of the fitness cost associated with non-susceptible pneumococcus[31,32].”

Line 110: any evidence re: the difference in exposure? Is this ok?

Thank you for this remark. If your comment refers to the sentence “Individuals are exposed to antibiotics independently of their colonisation status,” our approach was intended to capture the impact of antibiotic shortages on the ecology of the whole population, rather than only on prescribing in cases of confirmed pneumococcal infection. In France, amoxicillin is broadly prescribed for a wide range of common community infections, not limited to pneumococcal disease. For example, in diagnoses such as otitis, lower respiratory tract infections, unspecified diagnoses, or influenza, amoxicillin is prescribed in more than 50% of cases (ANSM, Report on antibiotic consumption 2000–2020 and Cheysson, Felix, et al., 2021). This

justifies our assumption that antibiotic exposure occurs independently of pneumococcal carriage. To clarify this point, we have expanded the relevant sentence in the manuscript as follows:

L. 128-131 "Individuals are exposed to antibiotics irrespective of their colonisation status, due to both high levels of asymptomatic pneumococcal colonisation and frequent antibiotic prescriptions for common community infections such as lower respiratory tract infections [21]"

Line 113: does this protection wane? Otherwise seems a strong assumption?

In our model, we assume that individuals exposed to antibiotics cannot acquire the sensitive strain of the corresponding antibiotic class. This is a standard assumption in models of antibiotic consumption and resistance. The protection does not persist beyond the treatment period: the effect of the antibiotic is assumed to last only for the duration of exposure (an average of 7 days for both beta-lactams and macrolides). This assumption seems biologically reasonable, as during treatment sensitive strains are rapidly cleared and therefore do not have sufficient time to establish themselves.

Line 114: based on descriptions elsewhere, can you clarify here that you don't actually model this infection but you calculate a fixed number of IPDs based on the number of colonized individuals?

Infections are not indeed a separate compartment in the model. This is a count of the number of infections based on the number of colonised individuals and a probability of infection. The calculation of this rate is detailed in the Methods section (L. 823-827) : "Invasive pneumococcal diseases" (IPD) included here meningitis and bacteremia. Assuming that carriage prevalence remained constant, we calculated the daily rate of developing invasive disease using IPD incidence a French national hospital-based laboratory network (Epibac), which reported 347 cases in 2021 for children under 5 years of age[58]. This yields an estimate of $\boldsymbol{\varepsilon_{IPD}}$= 4.8×10^{-7} days^{-1}.

We have reworded the sentence as follows:

L. 133-136 "Colonized children may develop invasive pneumococcal infections (IPDs). We computed expected IPDs by using a fixed rate of 4.8×10^{-7} per day during colonization, without explicitly modelling infections as a separate compartment."

Figure 2: why do s1 and s2 have such similar impacts? And why doesn't S3 reduce the DDD more than s1 / s2 ?

Thank you for this question and raising this confusion. We noticed that in Figure 1 the legend was incomplete, as we had inadvertently omitted the words "number of" before DDD/1,000/day. This may have caused confusion, and we have corrected it in the revised version.

Regarding why S3 does not reduce the number of DDD more than S1 or S2: in our simulations, all strategies were designed to be equivalent in terms of the overall quantity (in DDD) of antibiotics consumed. Consequently, in strategy S3, although the average prescribed daily dose is reduced, the number of treated individuals and the treatment duration remain unchanged. Therefore, the total number of amoxicillin DDD/1,000/day is similar across strategies for the same level of shortage.

The calculation is based on the generic formula: Number of DDD/1,000/day = (Volume of active substance sold × 1,000 inhabitants) / (DDD × study population).

In the case of amoxicillin DDD_{Amox}=1.5 (according to the WHO for a 1000mg tablet) and in the event of a 50% shortage

- For strategy S1: the number of exposed individuals is divided by 2:

$$NumberOfDDD/1{,}000_{Amox} = \frac{(NbExposedIndividual_{Amox}/2) * NbIntake * Dose * 1{,}000}{DDD_{Amox} * PopulationSize}$$

- For strategy S3: the administered dose is reduced from 1g to 0.5g (divided by 2).

$$NumberOfDDD/1{,}000_{Amox} = \frac{NbExposedIndividual_{Amox} * NbIntake * (Dose/2) * 1{,}000}{DDD_{Amox} * PopulationSize}$$

- For strategy S2: the duration of beta-lactam treatment is halved, which consequently reduces the exposure prevalence by half.

$NbIntake$= number of daily intakes (3 times daily for amoxicillin)

Since both strategies correspond to the same reduction in total active substance consumed, the total number of amoxicillin DDD/1,000 children/day is identical across S1, S2, and S3.

To ensure clarity for all readers, we have added an additional subsection (Supplementary Description 2) that provides a detailed explanation of these calculations.

Line 125: Could you specify / clarify that you refer / use pristinamycin as your example macrolide for the DDD calculation? And add to your methods. Is this commonly used in France? And an OK example macrolide?

Thank you for pointing this out. In France, pristinamycin and azithromycin are the two most commonly prescribed antibiotics within class J01F. For the DDD calculation and illustration in our analysis, we chose pristinamycin because its DDD value is of a similar order of magnitude to that of amoxicillin, which makes the comparison more readable. We could alternatively have chosen azithromycin; however, since its DDD is only 0.3 the resulting values would have been considerably less visible on the plot. It is important to note that this choice affects only the visualization in Figure 2. Since this part of the analysis is intended only as an illustration, the specific value chosen is not critical; the key point is the relative effect of the different strategies rather than the absolute scale.

Line 141: Should uncertainty in fitness costs be explored? I think it wasn't but could you comment in the discussion on how sure you are in your estimates of these? Variation here would have a big impact on the 1 yrs timings but I don't believe this is in your PRCC as it was a fitted parameter.

The reviewer is right in raising that point. The relative fitness is a poorly documented, important and debated parameter which may influence the conclusions in terms of dynamics and timing of resistance evolution.

In our model, relative fitness values were calibrated to reproduce real observed MIC resistance prevalence under antibiotic pressure. This calibration relies on the assumed initial MIC distribution among non-susceptible strains (informed based on French surveillance data), antibiotic-induced decolonization rates (fitted to two longitudinal epidemiological studies) and antibiotic exposure.

While resistance-associated fitness costs are biological parameters that should theoretically be stable across settings, we agree that exploring uncertainty around the fitted values is important. In response to your comment, we have now conducted an additional sensitivity analysis by varying the fitted relative fitness values by ±5% (Supplementary Figure 7). This analysis shows that such variations affect absolute outcome magnitudes but do not alter the relative ranking of shortage strategies. Since our analysis focuses on comparative rather than absolute impacts, our conclusions remain robust to this uncertainty.

More broadly, estimating resistance-associated costs remains challenging due to limited direct empirical data and reliance on indirect inference through compartmental model calibration, both representing ongoing limitations for mechanistic resistance modelling. We have expanded the Discussion to mention this, as follows :

L. 543-557 “[...] uncertainty in relative fitness values costs represents a key challenge, as these parameters are poorly documented and can influence resistance dynamics timings. In our model, relative fitness values were calibrated to reproduce observed MIC resistance prevalence under antibiotic pressure, based on the MIC distribution among non-susceptible strains (informed by French surveillance data), antibiotic-induced decolonization rates (fitted to longitudinal studies), and antibiotic exposure patterns. While resistance-associated fitness costs are biological parameters that should theoretically be relatively stable across high-income European settings, we conducted a sensitivity analysis varying the fitted values by ±5% to assess the impact of calibration uncertainty (Supplementary Figure 7). This analysis demonstrates that while such variations affect absolute outcome magnitudes, they do not alter the relative ranking of shortage strategies. Since our analysis focuses on comparative rather than absolute impacts, our main conclusions remain robust to relative fitness values uncertainty. More broadly, the estimation of resistance-associated costs remains challenging due to limited direct empirical data and reliance on indirect inference through compartmental model calibration, both representing ongoing limitations for mechanistic resistance modelling.”

Line 151: I struggle to understand this in terms of what this means for beta-lactam and macrolide use and then for Figure 3. Please clarify.

Thank you for this remark. We agree that our initial formulation was unclear. For scenarios S1, S2, and S3, the total quantity of antibiotic prescribed is the same regardless of the strategy (i.e. the number of DDD remains unchanged), but the distribution across individuals and doses differs. In contrast, for scenario S4, the situation is different: there is a reduction in the total quantity of beta-lactams prescribed and a corresponding increase in the quantity of macrolides. Because these belong to different antibiotic classes with different defined daily doses, the two cannot be directly compared in absolute quantities. To clarify this point in the revised manuscript, we have reworded the sentence as follows :

L. 171-174 “Strategies S1–S3 distribute the same overall quantity of antibiotics within the paediatric population but differ in terms of exposure patterns and prescribed doses, whereas strategy S4 involves a shift in antibiotic class, with fewer beta-lactams and more macrolides being prescribed.”

Line 155: Comparing between the strategies would be useful results to include here I think.

We thank the reviewer for this helpful suggestion. We have revised the text to highlight comparisons across strategies rather than describing each one separately, as follows:

L. 176-184 “Strategies S1 and S2 both decrease the prevalence of beta-lactam-exposed children as well as the number of amoxicillin DDD per 1,000 children and per day. However, S1 also reduces the incidence of beta-lactam-exposed children, while S2 leaves this incidence unchanged. In contrast, strategy S3 results in a decrease of the number of amoxicillin defined daily doses (DDD) per 1,000 children and per day, without affecting the incidence and prevalence of beta-lactam-exposed children. Strategy S4 leads to reductions in all beta-lactam-related outcomes and to an increase in the incidence and prevalence of macrolide-exposed children, as well as in the number of pristinamycin DDD per 1,000 children and per day.”

Line 165: in this paragraph could add something that all though some substantial percentage reductions the absolute levels don’t change enough to affect empiric prescribing decisions. Indeed the differences between the levels of resistance is quite minimal.

Thank you for this important comment. We acknowledge that our analyses focus on relative rather than absolute changes in resistance levels. As you note, the absolute differences observed are modest. Nonetheless, we consider that some of the strategies still produce meaningful shifts in resistance patterns, particularly for intermediate penicillin resistance (S3) and for macrolide resistance (S4). We believe that the strategies still provide insight into how prescribing policies may shift resistance patterns. We have clarified this point at the end of the paragraph as follows:

L. 201-202 “These findings mainly reflect relative rather than absolute changes in resistance levels, and the absolute differences remain modest.”

Line 216: I don’t understand why the linear trend is broken? Please discuss the underlying mechanism more here or in the discussion.

We agree with the reviewer that this result is not intuitive and deserves further discussion. The deviation from linearity arises because reducing treatment duration has two opposing effects: on the one hand, it lowers the selective pressure that favours resistant strains, but on the other hand, it also reduces the clearance of susceptible strains. These counteracting mechanisms balance each other and produce the non-linear pattern observed for strategy S3 (green curves). We have added a clarifying sentence in the Result to explain this point more explicitly :

L. 235-239 “This non-linear behaviour results from the combined influence of two opposing mechanisms: a decrease in selective pressure favouring resistant strains, alongside a reduction in the clearance of susceptible strains.”

Line 257: is this result discussed / explored in the discussion?

We thank the reviewer for highlighting the importance of discussing this result. While the statement on line 257 (“the daily dose reduction strategy S3 is never advisable”) reflects the French context, we agree that its applicability depends on country-specific settings. In the Results section, we interpreted the outcomes of strategy S3 in relation to differences between countries and highlighted that “the best strategy to “minimize the overall IPD incidence” is the daily dose reduction strategy S3 when macrolide resistance is initially more frequent than penicillin non-susceptibility ($MRSP^{init} > PNSP^{init}$), and the antibiotic switching strategy S4 otherwise ($MRSP^{init} \leq PNSP^{init}$)”. This information was previously presented in the Results

and Abstract only. In response to the reviewer's suggestion, we have now included it explicitly in the Discussion section as well :

L. 427-432 "Reducing the frequency of beta-lactam treatments in individuals appeared as the most effective strategy to manage pneumococcal resistance across Europe. Whereas the optimal strategy for managing IPD incidence varies between reducing beta-lactam daily dose and switching from beta-lactam to macrolides prescription."

Figure 5: please emphasise (add thick line or arrow?) which of these initial conditions is the "France" that you model in previous results.

Thank you for this pertinent suggestion. We have modified Figure 5 so that the parameters corresponding to the French data are now highlighted in bold.

Line 335: please make clear which parameters are "French" and which vary in this analysis

The detailed description of which parameters vary according to each country's context, and which remain fixed to those of the French analysis, is provided in the Methods section. To make this clearer in the Results, we have also added the following sentence:

L. 360-363 "The model was initialized with country-specific contexts reflecting their current levels of pneumococcal resistance and antibiotic, while other parameters were kept consistent with those used in the previous French analysis."

Figure 6: I don't understand what the numbers in this figure are. Variation over what? Is it just the "reduction" vs pre shortage 1 year earlier?

Thank you for your question. In this figure, the relative variation in resistance outcomes represents the proportional difference between the results after one year with a shortage and the results after one year without a shortage. For example, for PNSP this is calculated as:

$$\frac{PNSP_{After\ 1\ year\ of\ shortage} - PNSP_{After\ 1\ year\ without\ shortage}}{PNSP_{After\ 1\ year\ without\ shortage}} \times 100$$

Similarly, the variation in IPD incidence corresponds to the absolute difference in the number of cases per million children per year, comparing outcomes after one year with and without shortage. To clarify this point in the main text, we have added the following sentence in the Results section for Figure 6:

L. 363-366 "We analysed the results by computing the relative changes in resistance levels and absolute change in IPD incidence after a 1-year period of 50% beta-lactam shortage, compared to the same indicators after 1 year without shortages."

Line 347: between countries "and strategies" ?

Thank you for this suggestion. Our intention here is not to compare strategies with each other, but to compare countries for each given strategy. Indeed, we observed up to a threefold difference in IPD outcomes between Spain and the Netherlands, and this pattern is consistent across all strategies. We have clarified the text accordingly and completed the sentence as follows :

L. 374 "[…] between these countries for each strategy,"

Methods

Thank you for sharing the code.

Line 636: where does the 52% come from? And is “optimal” the correct word to use here and for the fitness cost values?

Thank you for these helpful remarks. We have now added in the main text the reference for the 52% value, which comes from household data collected in England. In addition, we agree that the term “optimal” was not appropriate in this context and have deleted it.

Line 638: what is “pneumococcus in otitis” and how did you use this data? This is linked to the major issue with not understanding your calibration methods?

Thank you for highlighting this ambiguity. We acknowledge that this point required clarification. “Pneumococcus in otitis” refers to pneumococcal isolates from children with acute otitis media by the French ACTIV research group (119 cases in 2021), available in the report of the National Reference Centre for Pneumococcus. These data were used to define the baseline distribution of penicillin minimum inhibitory concentrations (MICs) among resistant strains in 2021, which served as an input for our model calibration. Specifically, we combined the overall penicillin resistance prevalence observed in paediatric invasive pneumococcal disease (IPD) cases with the MIC distribution from otitis isolates to estimate the prevalence of each of the eight penicillin-non-susceptible resistance levels modelled. To clarify this point, we have expanded the Methods section as follows: .

L. 798-802 : “The prevalence of eight distinct penicillin-non-susceptible resistance levels was estimated as follows: the overall proportion of resistant strains observed in paediatric IPD cases was partitioned according to the distribution of MIC values documented in French paediatric otitis isolates from 2021 by the National Reference Centre for Pneumococcus.”

Line 663: Could you emphasise in this paragraph which is the one used in the main results? I think 50% reduction?

Thank you for this helpful remark. You are correct that the main results are based on a 50% reduction. While this information was already specified in the legends of all figures, we agree that it is useful to state it explicitly in the text. We have therefore added the following sentence in the Methods section:

L. 745-746 “In all the main results, a 50% reduction was applied across the different strategies and countries.”

Line 668: can you explain by how much S3 reduces the rate?

Thank you for this question. The detailed reductions applied to the decolonization rates under strategy S3 are provided in the Supplementary Materials. These reductions depend both on the level of resistance (as defined by MIC categories) and on the assumed percentage of reduction in each strategy.

Line 690: where are these reviewed studies from? France? How much variation in location might explain the variation seen?

Thank you for this comment. The duration of pneumococcal carriage was derived from a longitudinal study in Sweden conducted between 1995 and 2003 (before pneumococcal vaccine implementation), which reported a mean colonization period of approximately 43 days.

Carriage prevalence values came from four household studies in England conducted in 2001/2002, 2008/2009, 2012/2013, and 2015/2016, with a mean prevalence of 51.9% across all studies. The lower and upper bounds we report (32–51 days for duration; 35–57% for prevalence) correspond to coherent minimum and maximum values identified in the literature and were used only for sensitivity analyses to capture extreme scenarios.

We agree that carriage duration and prevalence can be influenced by socioeconomic conditions, healthcare access, and vaccine introduction, and may therefore vary across regions. However, our analysis focused specifically on European high-income countries with comparable vaccine coverage and healthcare infrastructure. We have clarified this in the Discussion:

L. 559-562 "[…]pneumococcal carriage prevalence, and duration of colonization, were held constant across countries based on European studies. These parameters are largely age-dependent and comparable across European high-income countries with similar healthcare systems"

Line 692: how did you define a "peak"?

Thank you for your remark. Indeed, the term "peak" was misleading here. What we meant is the mean (or central) value reported across the reviewed studies. We have rephrased this sentence to avoid ambiguity as follows :

L. 835-837 "duration of colonization in this age group ranged from 32 to 51 days, with a mean of 43 days[57–59], while the prevalence of carriage varied from 35% to 57%, with a mean of 52%[41,60]."

Line 694: does iteration number mean the number of samples? Perhaps change to that if so, or explain what iteration number means for here and line 709.

Thank you for your comment. You are correct, in the randomLHS function, n refers to the number of samples (rows) drawn, not to the iteration number. We have revised the manuscript to clarify this point as follows :

L. 838-840 "Parameter combinations were then sampled using Latin Hypercube Sampling (LHS), with 200 samples generated using the *randomLHS* function in the R package *lhs*."

L. 852-853 ". Parameter combinations were then sampled using LHS, with 500 samples generated using the *randomLHS* function in the R package *lhs*."

Line 696: was this just for France?

The sentence at line 696 (now line 840) refers to the uncertainty analysis, where we quantified uncertainty in model outcomes under shortage by varying assumed values for colonization duration and carriage prevalence: "For each new pair of parameters, the transmissibility rate was re-calculated."

To clarify: the uncertainty analysis itself was performed only for the French setting. However, the re-calibration of the transmission rate (β) was not limited to France. As described in the "Simulations of specific European countries" subsection, the transmission rate was re-calibrated for each country individually : "The calibrated fitness costs (f_k) were assumed to be equal to those calibrated from French data, while the transmission rate (β) was re-calibrated for each country."

Line 722: why only use 2021 data? Is there a trend in resistance / infection incidence before after? Is this ok in terms of COVID-19 impacts?

Thank you for this important question. Pneumococcal isolates from children with acute otitis media are only available every two years, and at the time of our analysis the latest data were from 2021. By 2021, the dynamics of pneumococcal resistance and invasive disease incidence had largely returned to pre-pandemic patterns, minimizing concerns about distortions due to COVID-19 (Pneumococcal National Reference Centre. 2023 Activity Report). Moreover, our objective was not to reproduce temporal trends in resistance over multiple years but rather to establish a representative steady state before the shortage, and then to evaluate the potential impact of a one-year shortage on pneumococcal ecology. We have added a clarifying sentence in the Discussion to address this point as follows :

L. 511-516 « […] the dynamics of pneumococcal resistance and invasive disease incidence may exhibit year-to-year fluctuations, our choice of 2021 as baseline (the most recent available data at the time of analysis) provides a representative post-pandemic steady state according to national surveillance[35], and our comparative framework focuses on relative impacts of shortage strategies rather than absolute temporal trends."

Line 728: does "tested" here mean those with drug susceptibility tests?

Yes, "tested isolates" refers to those for which antimicrobial susceptibility testing results were available. We have clarified this wording in the manuscript as follows :

L. 871-873 "We included European countries that had both antibiotic consumption data and antimicrobial susceptibility testing results for at least 30 tested isolates per year."

Discussion

- please add in something about how the changes in resistance prevalence are relatively minor (e.g. Figure 2b) and probably wouldn't affect empiric prescribing choices. As in resistance below 10% or above 20% wouldn't change what is prescribed or perhaps it would in France?

Thank you for this helpful suggestion. We have added sentences in the Discussion noting that the predicted changes in resistance prevalence are relatively minor and remain within ranges that would not modify empiric prescribing recommendations in France or most European settings. We also emphasize, however, that even small variations can still be relevant from a public health perspective. We have revised the manuscript as follows:

L. 562-568 "Such variations fall within the range of natural year-to-year fluctuations related to seasonality and serotype dynamics and would not modify empiric prescription recommendations. However, even small increases remain important from a public health perspective given both the severity and the preventability of IPD. Moreover, this estimate likely underestimates the true burden, as the model only accounts for changes in carriage prevalence and does not consider the potential for delays in treatment initiation or the use of less effective or less well-tolerated antibiotics during shortages, both of which could contribute to additional cases."

- line 391: do they really "vary widely"? I would context the "widely".

Thank you for your remark. We have added quantitative results to support our statement. For instance, in France, strategies S1 and S2 led to a decrease in multidrug resistance of 19.4%,

whereas S3 and S4 led to increases of 16.6% and 12.6%, respectively. Such contrasting directions of effect (substantial reductions under some scenarios versus marked increases under others) justify the use of the term “widely” when describing the variation in impact across strategies. We have revised the manuscript as follows:

L. 419-421 “For example, in France under a 50% shortage scenario, the estimated change in multidrug resistance ranged from a 19% decrease to a 17% increase, representing an overall spread of more than 35 percentage points across strategies.”

- line 400: can you mention the strategy names in this paragraph to link easily to the results? And expand acronyms on first use?

Thank you for this helpful suggestion. We have now included the names of the strategies in the first paragraph of the Discussion to facilitate the link with the Results section. In addition, all acronyms have been expanded upon first use, as recommended.

- line 400: can you add in a discussion of S3 too in this paragraph?

Thank you for this suggestion. The beginning of this paragraph does in fact refer to strategy S3. To make this clearer, we have explicitly indicated the name of the strategy, as also recommended in your previous comment :

L. 433-439” Reducing beta-lactam daily dose (strategy S3) was found to increase intermediate-resistant proportion at the population level due to the time-dependent action of the antibiotic. Indeed, lower defined daily doses have a lower efficacity against intermediate-resistant strains, allowing them to persist, as they are favoured over sensitive strains (which are easily eliminated) and resistant strains (which have a higher fitness cost). These findings are consistent with existing theoretical and observational studies showing that lower defined daily doses favour intermediate-resistant strains[22,23].”

- line 443: please expand on the lack of inclusion of emergence of resistance – is this ok for both beta-lactam and macrolide resistance? What are the mechanisms of that? How does this potentially bias your results? I’m thinking here about the differential impact of the strategies – fewer doses vs higher DDD may have different impacts on emergence.

Thank you for this important remark. Our model does not account for the de novo emergence of resistance. Over a one-year horizon in children, in a context where resistance levels are already significant, the contribution of de novo emergence is negligible compared with transmission. Excluding emergence could theoretically underestimate the impact of reduced dosing (strategy S3), since in other bacteria suboptimal drug exposure may favour resistance within the “mutant selection window”. However, evidence indicates this process plays only a minor role for *S. pneumoniae* and we thus expect resistance dynamics to be driven primarily by selection and spread of existing resistant clones

- line 494: could IPD even have been reduced / not affect with the right antibiotic usage?

Thank you for raising this interesting point. In principle, if all high-risk children were systematically prioritized for first-line antibiotics, the predicted impact of shortages on IPD might be negligible. In practice this scenario is unlikely to be fully realised, as identification of high-risk cases is not always immediate. Moreover, dispensing delays and stock fragmentation

occur, and the use of second-line or less effective antibiotics is more likely during shortages, which can contribute to additional cases of IPD even under prioritisation. We now expand on this question in the Discussion, as follows:

L. 532-535 "[…], although the predicted changes in IPD would be smaller, assuming perfect identification and prioritization of high-risk cases. In practice, delays in risk assessment, stock fragmentation, and increased use of less effective alternatives during shortages would likely still result in increased IPD incidence."

- line 512: could you comment on which strategies are potentially implementable? And how they would be enforced? Is it likely that policymakers could recommend one strategy or would a combination be the most likely to happen?

Thank you for this useful comment. In terms of feasibility, reductions in prescription frequency (S1) and treatment duration (S2) are the most realistic strategies, since they are already consistent with antibiotic stewardship recommendations. Importantly, such measures are better implemented as part of routine prescribing practice rather than enforced suddenly during shortages, which could otherwise risk undertreatment of infections. Reducing daily dose (S3) represents the least acceptable and most theoretical scenario, because of concerns about treatment efficacy. While switching to macrolides (S4) is not desirable from a resistance perspective, it is likely to occur in practice during shortages in order to ensure that infected children can still be treated. In reality, a policy response would probably consist of a combination of S1 and S2 as sustainable strategies, while S4 would represent a pragmatic but less desirable, response in the context of acute shortages. We have clarified this in the revised Discussion :

L. 636-647 "Among the four strategies, reductions in antibiotic frequency (S1) and treatment duration (S2) are both feasible and aligned with good stewardship practice, though ideally such measures should be applied systematically rather than suddenly enforced during shortages to avoid undertreatment of infections. Reducing daily dose (S3) appears the least acceptable and most theoretical scenario, whereas switching to macrolides (S4), although undesirable for resistance, would likely occur in practice during shortages to ensure treatment of infected children. In reality, policy responses would most plausibly rely on a combination of S1 and S2, with S4 acting as a pragmatic but suboptimal response during acute shortages."

- line 519: This paragraph needs a "so what?". Is 4 ok? is this within annual fluctuation? how many IPD lead to death etc? Any decisions made on this in the past?

We agree that more insight on the public health consequences of our results was needed at this point of the Discussion. We have added the following section to this paragraph:

L. 575-588 ". In the latter interpretation, strategy S1 can be used to estimate the epidemiological cost of shortages, with an additional increase of 1 to 3 IPD per 1 million children per year for a 50% shortage across European countries. This corresponds to a relative increase of 0.6–4.3% compared with the reported European incidence of IPD in children under 5 (70–150 cases per million in 2021[48]). With an estimated case fatality rate of 3.6%[48], this translates into fewer than one additional paediatric death per million children annually. Although such variations fall within the range of natural year-to-year fluctuations related to seasonality and serotype dynamics, even small increases remain important from a public health perspective given both the severity and the preventability of IPD. Moreover, this estimate likely underestimates the true burden, as the model only accounts for changes in carriage prevalence and does not consider the potential for delays in treatment initiation or the use of

less effective or less well-tolerated antibiotics during shortages, both of which could contribute to additional cases.”

- line 536: linked to the major comments, I think here more needs to be made on amoxicillin resistance vs macrolide resistance vs dual resistance and which is more concerning and why.

Thank you for this constructive comment. To address it, we have expanded the discussion section to explicitly compare amoxicillin resistance, macrolide resistance, and dual resistance. We now highlight that amoxicillin resistance directly compromises the most widely used first-line therapy, macrolide resistance has been recognized as a global concern by WHO (2024), and dual resistance represents the most critical scenario as it leaves very limited therapeutic options. This new paragraph clarifies the clinical and public health implications of each type of resistance and emphasizes why MDR constitutes the greatest challenge. As addressed in our response to your earlier comment (in the major revision part), we revised the Discussion as follows :

L. 593-602 “It is important to distinguish between the implications of penicillin-resistance, macrolide-resistance, and dual resistance. Amoxicillin is the first line and most affordable option for pneumococcal infections; resistance therefore directly affects standard care. Macrolide resistance, though less critical in some situations, is still recognized as a major global issue; *S. pneumoniae* resistant to macrolides was recently included by WHO (2024) among its priority pathogen–antibiotic combinations. Dual resistance to both beta-lactams and macrolides remains the most worrisome scenario, as it leaves only fluoroquinolones or newer, less available agents as options. This situation increases costs, safety risks, and the likelihood of further resistance selection, making MDR the greatest therapeutic and public health challenge. Importantly, infections caused by resistant strains are associated with higher mortality[49].”

- line 540: up to s4?

Thank you for this observation. You are correct, S4 should be included as it represents a strategy relevant to antibiotic stewardship initiatives more broadly, particularly those supporting antibiotic cycling or switching to mitigate antimicrobial resistance. We have revised the sentence (line 618) accordingly to read "our explored strategies (S1-S4)".

- line 555: Can you make any conclusions on whether antibiotic shortages are overall bad? and in what way? As in, could they in fact reduce antibiotic resistance? Can you comment on the overall likely effect.

Thank you for raising this important point. Our results indeed suggest that shortages can reduce antibiotic exposure and thereby slow resistance evolution at the population level. However, this apparent “benefit” comes at the cost of increased burden of invasive pneumococcal disease and likely higher morbidity and mortality in children. Beyond these clinical issues, shortages also carry an economic burden, since the use of second-line agents such as macrolides is typically more expensive, and poorly treated infections often require additional healthcare resources, including hospitalisation. Overall, shortages cannot be considered beneficial: the unintended reduction in resistance is outweighed by the immediate clinical and economic costs. Instead, the results emphasize that the desirable effect of reduced antibiotic use should be pursued through deliberate stewardship interventions, not uncontrolled antibiotic shortages. As addressed in our response to your earlier comment (in the major revision part), we revised the Discussion as follows :

L. 661-669 “Although our model suggests that antibiotic shortages may reduce the selective pressure driving resistance, this apparent benefit is outweighed by an increased burden of severe infections and contributes to preventable morbidity and mortality in children. Shortages also carry an additional economic cost, as second-line alternatives such as macrolides are generally more expensive, and poorly treated infections often lead to increased healthcare utilization, including hospital admissions. Overall, shortages cannot be considered beneficial: the unintended reduction in resistance is outweighed by the immediate clinical and economic costs. Instead, the aim of reducing antibiotic use should be achieved through controlled stewardship strategies rather than through unpredictable shortages.”

Second return to reviewers

REVIEWER COMMENTS

Reviewer #2 (Remarks to the Author):

Thank you for addressing all comments and questions from reviewers.

Reviewer #2 (Remarks on code availability):

The code is sufficiently documented

Reviewer #3 (Remarks to the Author):

I co-reviewed this manuscript with one of the reviewers who provided the listed reports. This is part of the Nature Communications initiative to facilitate training in peer review and to provide appropriate recognition for Early Career Researchers who co-review manuscripts.

Reviewer #3 (Remarks on code availability):

Not applicable

Reviewer #4 (Remarks to the Author):

Thank you for these revisions. They are mostly highly appropriate and I think have strengthened the manuscript. However I have two remaining issues.

Thank you for this positive comment.

Firstly, a minor point that care still needs to be taken on the fitness cost - line 737 this is relative fitness not cost?

We thank the reviewer for pointing this remaining mistake out. In response to your previous review, we changed "fitness cost" to "relative fitness" throughout the manuscript, since f_k does indeed represent relative fitness mathematically. However, we inadvertently missed this correction at line 737. It has now been corrected.

Secondly, the calibration is still unclear (line 776 onwards). This just needs a few minor tweaks.
- How were beta and f_k “calibrated”? by hand?

In both steps 1 and 2, the transmission rate (β) and relative fitness (f_k) are numerically calibrated to ensure that model-predicted carriage prevalence and resistance frequency remain stable over time at the initial conditions, under community-level antibiotic exposure. This calibration is not performed by hand but rather through the minimization of the the least-squares distance between model predictions and observations using the Limited-memory BFGS (L-BFGS) optimization algorithm with box constraints (optim function in R).

During step 1 (estimation of antibiotic-induced decolonization rates from clinical studies), for each candidate set of decolonization rates, we re-estimate β and f_k to ensure that carriage prevalence and resistance frequency remain at steady-state. Note that these values are only used locally to enable estimation of antibiotic effect calibration, as the relative fitness and beta are calibrated later in step 2.

Once the antibiotic-induced decolonization rates are determined from step 1, we fix these values and estimate β and f_k for the main model (step 2). Specifically, we fix antibiotic consumption at the French 2021 level and calibrate β and f_k so that model-predicted carriage prevalence and resistance frequency (expressed as MIC distribution) match the 2021 observed French surveillance data.

To clarify that this was an automated optimization procedure and not performed by hand, we have revised the relevant sections describing both calibration steps (L. 770-786 and L. 796-807) and added the following explicit description of the optimization method:

L. 812-817 “All parameter estimations in both calibration steps were based on least-squares minimizations, performed running the Limited-memory BFGS (L-BFGS) optimization algorithm that approximates the Broyden-Fletcher-Golfarb-Shanno algorithm using box constraints[43]. The algorithms were implemented using the optim function from the *stats* R package. The optimization procedure continued until convergence, defined as the point at which the relative reduction in the objective function between successive iterations fell below the algorithm tolerance threshold of 10^{-9}.”

- Which of the parameters in Supplementary table 1 did you “fit to”? I think it is the “after treatment” carriage and resistant proportion but this is not totally clear. Which of the parameters in this table are you able to include in your model (e.g. population size? Age?) and which are parameters from the data there just for information?

We thank the reviewer for this important comment. To clarify, we used a nested optimization procedure where different data from two clinical studies, reported in Supplementary Table 1, were used at each step. First, we fitted the transmission rate (β) and relative fitness values (f_k) to the initial carriage prevalence and initial resistance proportion (pre-treatment data from the two studies), running the model for five years to establish a steady state (using colonization duration and antibiotic exposure rates informed from the literature). Then, to estimate the antibiotic-induced decolonization rates, we fitted to the after-treatment carriage prevalence and after-treatment resistance proportion from these same studies, using treatment duration data from the studies. Population size and age distribution are provided in Supplementary Table 1 as descriptive characteristics of the study populations but were not used as model parameters. Our model is deterministic and not age-stratified; hence these demographic variables do not directly inform model parameterization**.**

To address this comment and the following one, we have restructured Supplementary Table 1 and revised its title to clarify what is reported in the table, as follows:

	**Amoxicillin-clavulanate (40 and 5.7mg/kg/d, respectively, in two divided doses)**	**Erythromycin/sulfisoxazole (50 and 150mg/kg/d, respectively, in three divided doses)**	**Sources**
**Descriptive data on the clinical studies on antibiotic exposure impact**			
Population size	146	86	Amoxicillin study[1] Erythromycin *study* [3]
Age of the population	6-35 months	3-36 months	
**Literature-derived parameters used for model calibration**			
Duration of colonization $(1/\lambda)$ (days)	43	43	Högberg et al.[7] Ekdahl et al.[8]
Antibiotic exposure rate (Φ) /hab/day	0.007	0.002	Guillemot et al.[9]
**Data from the clinical studies used for model calibration**			
Initial carriage prevalence	0.64	0.47	Amoxicillin study[1] Erythromycin *study* [3]
Initial resistance proportion	0.19	0.53	
After treatment carriage prevalence	0.13	0.25	
After treatment resistance proportion	0.67	0.91	
Duration of treatment $(1/\gamma)$ (days)	7	8	
**Calibrated parameters on antibiotic-induced decolonization**			
Rate of antibiotic-induced decolonization rate on susceptible strains	$\delta_{PSSP}^{Amox} = 1$	$\delta_S^{Macro} = 1$	Calibrated
Rate of antibiotic-induced decolonization rate on resistant strains	$\delta_{PNSP}^{Amox} = 0.090$	$\delta_R^{Macro} = 0.035$	

Supplementary Table.1 Characteristics of studies used to calibrate antibiotic-induced decolonization rates, model parameters used for calibration, and resulting calibrated decolonization rates.

This in unclear between line 800 and then 802 where it says that you use levels from "before antibiotic exposure"?

We agree that this was unclear. As we are using two clinical studies where cohorts of children exposed to a single antibiotic course were followed up during their treatment, by "before antibiotic exposure", we referred to the situation before this specific exposure. This does not mean that the associated carriage and resistance levels corresponded to expected levels in the absence of antibiotics; rather, they reflected epidemiological conditions in the community, as in these two studies children were selected from the community. Then, the values after the

antibiotic treatment exposure correspond to carriage and resistance levels just after the end of the treatment in the selected group of children who were all given treatment. This allowed us to capture the direct effect of antibiotic effect on decolonization rate in susceptible and resistant.

We have clarified this as follows:

L. 774-778: "For each candidate set of decolonization rates, the model was initialized at a stable pre-treatment baseline resulting from typical community antibiotic exposure. To reproduce this baseline, the transmission rate β and relative fitness vales f_k were calibrated so that carriage prevalence and resistance frequency remained stable over five years, under community-level antibiotic exposure."

- Supplementary table 1: what is "calibrated data"? do you mean "estimated parameters"?
- I would suggest that you split supplementary Table 1 into

o Study information

o Data levels fitted to or something

o Fitted parameters (not data)

We thank the reviewer for this suggestion. We agree that Supplementary Table 1 lacked clarity in its original format. We have now restructured it into distinct sections as recommended: (i) Descriptive data on the clinical studies on antibiotic exposure impact, (ii) Literature-derived parameters used for model calibration, (iii) Data from the clinical studies used for model calibration, and (iv) Calibrated parameters on antibiotic-induced decolonization. We believe this new organization presents the information more clearly.

- What is the objective function? (line 798)

Thank you for this question. We have now reformulated the calibration part and the sentence "We repeated this two-step process until convergence, defined as when the relative reduction in the objective function fell below 10^{-9}, to identify the antibiotic-induced decolonization rates that minimized the distance between predicted and observed carriage prevalence and resistance frequency at the end of treatment." you were referring to has been deleted from the text. The objective function we were referring to on line 798 was the mean squared distance between model-predicted and observed carriage prevalence and resistance frequency at the end of treatment, and the corresponding antibiotic-induced decolonization rates were those that minimized this distance. The entire calibration process has been clarified, as described earlier.

- Line 818: I am still not 100% sure what the observed values were – overall proportion of resistant strains with the 8 MIC values in Table 1? Could you just link the sentences in 818-822 more clearly so that the "eight" clearly links to the "distribution of MIC values" perhaps linked to those shown in Table 1 and that these levels are the observed values?

The observed values used for calibration in step 2 were : the carriage prevalence, the distribution of penicillin-non-susceptibility across 8 MIC levels and the proportion of macrolide resistance in 2021. Table 1 presents the calibrated parameter values (antibiotic-induced decolonization rates and relative fitness values), not the observed resistance distributions. The observed 2021 resistance distribution values used as calibration targets are provided in Supplementary Table 2.

To clarify this distinction and ensure that the link between the eight MIC levels and the observed distribution is explicit, we have reworded the relevant sentences as follows:

L. 796-807 "In the second stage, we fixed antibiotic consumption frequency at the French 2021 level and fixed the decolonization rates at the values calibrated from the clinical studies above (step1). Assuming that carriage prevalence and resistance distributions have remained stable since 2021, we calibrated β and f_k to ensure that both carriage prevalence and resistance levels for macrolide and penicillin resistance levels k remained stable over a five-years period, matching the observed 2021 values (pre-shortage equilibrium). Optimization was performed by minimizing the distance between simulated data over five years and the observed 2021 carriage prevalence and resistance distributions. Penicillin-non-susceptibility was characterized using 8 MICs levels (0.063, 0.125, 0.25, 0.5,1,2,4,8 mg/L), and macrolide resistance using 2 levels. The 2021 distribution of MIC values for penicillin resistance was obtained from French paediatric otitis isolates data, documented by the National Reference Centre for Pneumococcus (see Supplementary Table.2)."

- Line 830 – how was the optim function used in the two step first process? As mentioned above was the calibration partly by hand?

The calibration was not done by hand; it was fully automated. We believe that the wording "two-step first process" (line 797) was misleading, because these computations were not performed as two separate sequential steps. Instead, they were carried out within a single routine in which one optimization is called inside the other.

To estimate the antibiotic-induced decolonization rates, we used the L-BFGS-B algorithm to estimate the values that best matched the observed changes in carriage prevalence and resistance frequency after antibiotic treatment in clinical studies. However, for values of antibiotic-induced decolonization rates tested by the algorithm, the model needed to start from realistic initial conditions, specifically, a stable baseline matching the pre-treatment carriage prevalence and resistance frequency observed in each study. To achieve this, we used a nested optimization: for every candidate evaluated, we automatically ran a second optimization to re-estimate β and f_k so that the model reached the correct baseline steady state. This nested structure means that the estimation occurs simultaneously within the same optimization loop, not as separate sequential steps. Thus, β and f_k are not calibrated by hand; they are recalibrated automatically for every candidate decolonization rate during the optimization. We have clarified this procedure in the revised manuscript as follow :

L. 770-786 "For each study, we calibrated the model to estimate the antibiotic-induced decolonization rates that best reproduced the observed changes in carriage prevalence and resistance frequency following antibiotic treatment in children from the study. Calibration was performed by minimizing the distance between predicted and observed carriage prevalence and resistance frequency at the end of treatment using an optimization algorithm. For each candidate set of decolonization rates, the model was initialized at a stable pre-treatment baseline resulting from typical community antibiotic exposure. To reproduce this baseline, the transmission rate β and relative fitness vales f_k were calibrated so that carriage prevalence and resistance frequency remained stable over five years, under community-level antibiotic exposure. These β and f_k estimates were used only to support calibration at this stage, as both parameters were re-calibrated later in step 2. We applied a nested optimization procedure: for each candidate decolonization rate, a secondary optimization automatically re-calibrated β and f_k to ensure that the model reproduced the correct pre-treatment steady state. Final estimates were: $\delta_{PNSP}^{Amox} = 0.09\, days^{-1} and\ \delta_{PSSP}^{Amox} = 1\, days^{-1}$ and $\delta_{R}^{Macro} = 0.035\, days^{-1}\ and\ \delta_{S}^{Macro} = 1\, days^{-1}$. The pre-treatment and post-treatment carriage and

resistance data from both the Finnish and French studies used for calibration are provided in Supplementary Table.1. The resulting calibration estimates are shown in Supplementary Figures 10 and 11."

It is great to see the new sensitivity analysis and thanks for pointing out the importance of relative not absolute in your results.

We thank the reviewer for this positive feedback and for the constructive suggestions that led to these improvements in the manuscript.

Unfortunately I wasn't able to reproduce your results with the existing code - please provide more structure and check the dependencies.

We thank the reviewer for bringing this to our attention and apologize for the initial difficulties in running the code. We have thoroughly tested the code and resolved the reproducibility issues. Additionally, we have enhanced the documentation by providing clearer instructions on the execution order of the R files and their dependencies. The code should now be fully reproducible.

Reviewer #4 (Remarks on code availability):

Although some packages are set up initially, the internal code needed many more packages (e.g. rayshadr, triangle)

And then this file was missing or rather the setup pointed to something on the author's computer but I don't have it: gen <- odin::odin("C:\\Users\\maurinau\\OneDrive - LECNAM\\Stage\\StagePasteur\\codeRStage\\DeuxAgeDeuxResistanceOdin.R")

The authors need to provide more structured guidance on the order and way to run the R / Rmd file and check the file address dependencies.

I could not reproduce the work in the current state - using the here package and giving me more instructions on how to use the scripts would enable this. A lot of the code is here which is great but more commenting and support is needed.

We apologize for the reproducibility issues and thank the reviewer for the detailed feedback. We have made the following improvements to address all the concerns raised:

Package dependencies: We have now documented all required packages in the README file on GitHub.

File paths: all absolute paths have been replaced with relative paths using the here package, eliminating system-specific path issues.

Code structure and execution order: we have added a dedicated section in the README providing clear, step-by-step instructions on the order in which to run the R files.

Important clarification regarding Rmd files: the Rmarkdown files in our repository are used solely for code organization and should not be knitted/compiled into output documents.

The code should now be fully reproducible. We appreciate the reviewer's valuable suggestions for improving code accessibility.

Third round of feedback to reviewers

REVIEWER COMMENTS

Reviewer #4 (Remarks to the Author):
Thank you - comprehensive response, nothing else from me on the text.

Reviewer #4 (Remarks on code availability):

I cannot run the code - I get multiple errors.
Initially in the first RMd I think line 102 should not be "Optim" but Optimi with an i?
The first two files then fun but then I cannot run the UndertaintyAnalysisFigure1 as I do not have "rayshader" - its not in the list of needed installations at the start and then, when I try to install from "install.packages" it fails and I get "OpenGL is not available in this build" etc.

Please double check with a clean computer set up that this all works. I'm happy to try this again once you have had another look. Please also include headings and descriptions of what each file does.

We thank the reviewer for testing the code and reporting these issues. We fully agree that the code must be reproducible and have carefully addressed all the points raised.

As suggested, we re-ran the entire code on a clean computer without any pre-installed packages. Through this process, we identified that some packages required specific versions to work with this version of R, and we removed packages that were not necessary for the analysis.

The latest version of the code is now available on the GitHub repository, along with detailed instructions for package installation. The required R version (4.3.0) and Rtools (4.3) are specified in the documentation.

We have tested the full pipeline on two independent computers and confirmed that it runs without errors.

Additionally, as requested, we have included headers and descriptions in each R file. A detailed description of what each file does is also provided in the README section of the GitHub repository.

We hope this updated version runs smoothly and remain available for further testing.
